# Steering cell migration by alternating blebs and actin-rich protrusions

**DOI:** 10.1186/s12915-016-0294-x

**Published:** 2016-09-02

**Authors:** Alba Diz-Muñoz, Pawel Romanczuk, Weimiao Yu, Martin Bergert, Kenzo Ivanovitch, Guillaume Salbreux, Carl-Philipp Heisenberg, Ewa K. Paluch

**Affiliations:** 1 Max Planck Institute of Molecular Cell Biology and Genetics, Dresden, 01307 Germany; 2 International Institute of Molecular and Cell Biology, Warsaw, 02-109 Poland; 3 Cell Biology and Biophysics Unit, European Molecular Biology Laboratory, Heidelberg, 69117 Germany; 4 Max Planck Institute for the Physics of Complex Systems, Dresden, 01187 Germany; 5 Department of Biology, Institute of Theoretical Biology, Humboldt University, Berlin, 10115 Germany; 6 Institute of Molecular and Cell Biology, A-STAR, Singapore, 138673 Singapore; 7 Medical Research Council Laboratory for Molecular Cell Biology, University College London, WC1E 6BT London, UK; 8 The Francis Crick Institute, Lincoln’s Inn Fields Laboratories, 44 Lincolns Inn Fields, London, WC2A 3LY UK; 9 Institute of Science and Technology Austria, Klosterneuburg, 3400 Austria; 10 Present address: Department of Cardiovascular Development and Repair, Centro Nacional de Investigaciones Cardiovasculares (CNIC), 28029 Madrid, Spain

**Keywords:** Cell migration, Protrusion orientation, Directionality, Persistence, Bleb, Actin-rich protrusion, Run and tumble

## Abstract

**Background:**

High directional persistence is often assumed to enhance the efficiency of chemotactic migration. Yet, cells in vivo usually display meandering trajectories with relatively low directional persistence, and the control and function of directional persistence during cell migration in three-dimensional environments are poorly understood.

**Results:**

Here, we use mesendoderm progenitors migrating during zebrafish gastrulation as a model system to investigate the control of directional persistence during migration in vivo. We show that progenitor cells alternate persistent run phases with tumble phases that result in cell reorientation. Runs are characterized by the formation of directed actin-rich protrusions and tumbles by enhanced blebbing. Increasing the proportion of actin-rich protrusions or blebs leads to longer or shorter run phases, respectively. Importantly, both reducing and increasing run phases result in larger spatial dispersion of the cells, indicative of reduced migration precision. A physical model quantitatively recapitulating the migratory behavior of mesendoderm progenitors indicates that the ratio of tumbling to run times, and thus the specific degree of directional persistence of migration, are critical for optimizing migration precision.

**Conclusions:**

Together, our experiments and model provide mechanistic insight into the control of migration directionality for cells moving in three-dimensional environments that combine different protrusion types, whereby the proportion of blebs to actin-rich protrusions determines the directional persistence and precision of movement by regulating the ratio of tumbling to run times.

**Electronic supplementary material:**

The online version of this article (doi:10.1186/s12915-016-0294-x) contains supplementary material, which is available to authorized users.

## Background

Efficient directed migration is assumed to rely on high directional persistence [1–3]. Indeed, in a stable chemotactic gradient, straight trajectories allow to reach the target in a minimal time. In contrast, lower directional persistence has been associated with poorly directed migration such as in the absence of chemotactic cues or in shallow chemotactic gradients [2, 3]. For instance, the persistence of fibroblasts and dendritic cells has been shown to decrease in presence of a uniform concentration of chemoattractant when compared to migration of the same cells in a chemotactic gradient [4]. Yet, cells undergoing directed migration in vivo often display trajectories with frequent direction changes and low persistence compared to directed migration in culture [5–7]. Such trajectories have been described as biased random walks or as series of runs and tumbles, i.e., alternating phases with high and low directional persistence [8–11]. In zebrafish primordial germ cells, whose chemotactic migration during development can be described as a succession of run and tumbles, low persistence and frequent direction changes associated with tumbling have been proposed to fine-tune the migration of these cells, as they progress to intermediate targets during development [9, 12]. However, the cellular mechanisms controlling directional persistence during animal cell migration in vivo are poorly understood, and the functional importance of a proper control of this parameter remains elusive.

Here, we investigate the cellular control and function of directional persistence during cell migration in vivo. We use zebrafish early mesendoderm progenitor cells, which, during early gastrulation, predominantly migrate as single cells and display frequent direction changes [6]. We have previously shown that mesendoderm progenitors can form different protrusion types, including blebs and actin-polymerization driven ones, and that enhancing the formation of blebs decreases migration directional persistence [13]. Therefore, we reasoned that mesendoderm progenitors represented a good model for investigating migration directionality in vivo.

We first show, using an unbiased trajectory analysis algorithm, that lateral progenitors migrating towards the forming body axis alternate run and tumbling phases. We then employ a transplantation assay to investigate how protrusion formation relates to migration directionality during single cell migration of progenitor cells. Using custom-made cell segmentation and protrusion detection software, we show that run phases correlate with the formation of directed actin-rich protrusions, while enhanced blebbing is observed during tumbles. Changing the proportion of blebs to actin-rich protrusions changes the ratio of tumbling to run times. Strikingly, we observe that both decreasing and increasing the ratio of tumbling to run times increase cell dispersion during migration, indicative of reduced migration precision. A theoretical model quantitatively recapitulating the characteristics of progenitor cell migration indicates that an optimal tumbling-to-run ratio enhances migration precision in a changing environment. Together, our experiments and model suggest that the precision of mesendoderm progenitor cell migration depends on the ratio of tumbling to run times, and that this ratio is controlled by adjusting the proportion of blebs to actin-rich protrusions formed by these cells.

## Results

### Zebrafish lateral mesendoderm progenitors display run-and-tumbling during directed migration

**Fig. 1:**
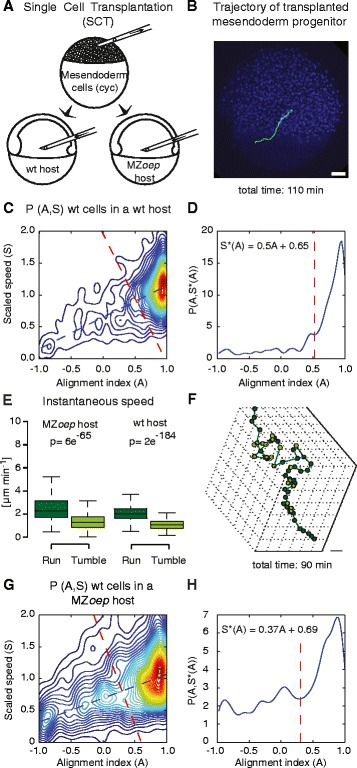
Mesendodermal cells display runs and tumbles during directed migration. **a** Schematic of the single cell transplantation experiments where mesendoderm progenitor cells are transplanted into a wt or MZ*oep* host. **b** Lateral view of a host embryo (ectodermal nuclei are labeled with Histone-Alexa 647 in blue) at 60 % epiboly (7hpf) with an example track of a control (green) mesendoderm cell transplanted into the lateral germ ring margin at 50 % epiboly (5.5hpf). Scale bar = 50 μm. **c** Two-dimensional probability density of the alignment index (A) and scaled speed (S), P(A,S), calculated for mesendodermal cells transplanted into wt hosts (*n* = 18). The blue dashed line shows the linear fit to the maximum values of P(A,S) for A. The red dashed line is the line, perpendicular to the maximum, defining the threshold above which a portion of a trajectory is considered to be a run phase (also in **d**). The intersection point is at A = 0.52, corresponding to the local minimum between the global maximum and the nearest local maximum of P(A,S) along the maximum line (displayed in **d**). **d** One-dimensional cross-section of P(A,S) along the maximum line, S*(A). **e** Instantaneous speed of single mesendoderm cells transplanted into wt and MZ*oep* hosts during run and tumble phases. N = 854 runs and 478 tumbles in MZ*oep* hosts (23 cells) and 1317 runs and 484 tumbles in wt hosts (18 cells). Statistical significance by t-test. **f** Exemplary three-dimensional cell trajectory displaying run (dark green) and tumbling phases (light green). The points represent cell positions over time. Scale bar = 50 μm. **g** Two-dimensional probability density P(A,S), calculated for mesendodermal cells transplanted into MZ*oep* hosts (*N* = 23). Lines as in c. The intersection point is at A = 0.3. **h** Like “**d**” for probability density in “**g**”

In order to investigate how migration directionality is determined in zebrafish mesendoderm progenitors, we transplanted mesendodermal cells (cells expressing the Nodal-ligand Cyclops (Cyc), to induce mesendoderm cell fate [14]) injected with a fluorescent histone in a wild type (wt) host (Fig. 1a). The transplanted cells displayed mostly single cell migration, with only sporadic interaction with neighboring mesendoderm progenitors, for at least 3 hours following transplantation (from 30 min before shield to 70 % epiboly), as previously reported [6]. Cell nuclei were tracked for over 2 hours during mid gastrulation stages (~6–8 hours post-fertilization (hpf), starting 30 min to 1 hour post-transplantation) (Fig. 1b). We found that the trajectories of transplanted mesendoderm progenitors displayed a mean persistence, i.e., ratio of the net displacement to cell trajectory length, of 0.68 ± 0.13 (mean ± standard deviation (SD), *n* = 18 cells), lower than the typical persistence values observed during chemotaxis in vitro [15, 16]. An unbiased analysis of the trajectories’ cell scaled speed (S) and alignment index (a measure of the local persistence, A) revealed that the cells displayed a multi-modal behavior that can be described as alternating phases of relatively straight migration (run phases) and phases of slowed and poorly directed movement (tumble phases). Accordingly, the cell trajectories could be divided into run and tumble phases, where the cut-off between phases was determined automatically, based on a quantitative analysis of the local persistence and speed of the cells (Fig. 1c, d and Additional file 1: Supplementary Methods for details). This automated analysis yielded an average ratio of tumbling to run times in mesendodermal progenitors of 0.58 ± 0.34 (mean ± SD, *n* = 18 trajectories). The relatively large SD reflects the fact that both run and tumble times displayed exponential distributions, which are characterized by SDs of the order of the mean (Additional file 2: Figure S1). Instantaneous cell speed, measured with a 1.5 min time interval, was approximately 1.8 times higher during run phases compared to tumble phases (Fig. 1e). Finally, tumbles usually resulted in a significant direction change, with an average angle between successive runs of 56 ± 34 degrees (mean ± SD, *n* = 18 trajectories).

Even though lateral progenitors display mostly single cell migration in early gastrulation [6], they still transiently interact with neighboring mesendoderm progenitors, which could influence their trajectories. To investigate the migration of these cells in an in vivo environment while avoiding any influence of transient contacts with neighboring cells, we transplanted single mesendoderm cells, into the lateral side of maternal zygotic *oep* (MZ*oep)* mutant embryos, which lack mesendoderm progenitors [17]. Transplanted cells display directed migration between the yolk and the overlying ectoderm towards the dorsal side of the embryo, as their wt counterparts, but do not have neighboring cells to interact with [5]. Thus, they represent a good model system for the study of single cell migration in a complex in vivo environment. We acquired trajectories of mesendoderm progenitors injected with a fluorescent histone transplanted into MZ*oep* hosts and applied the same automated analysis as described above to their trajectories. We found that, similarly to progenitors transplanted into wt hosts, the cells displayed multi-modal trajectories that can be described as successions of run and tumble phases (Fig. 1f–h). Similar to progenitors migrating in wt hosts, the average ratio of tumbling to run times was 0.68 ± 0.38 (mean ± SD, *n* = 23 trajectories), instantaneous cell speed was approximately 1.8 times higher during run phases compared to tumble phases (Fig. 1e), and tumbles resulted in a significant direction change, with an average angle between successive runs of 68 ± 37 degrees (mean ± SD, n = 23 trajectories).

Taken together, our analysis indicates that zebrafish mesendoderm progenitors alternate phases of directed migration (runs) and reorientation events (tumbles) during directed migration in vivo.

### Protrusion formation during run and tumbling phases

**Fig. 2:**
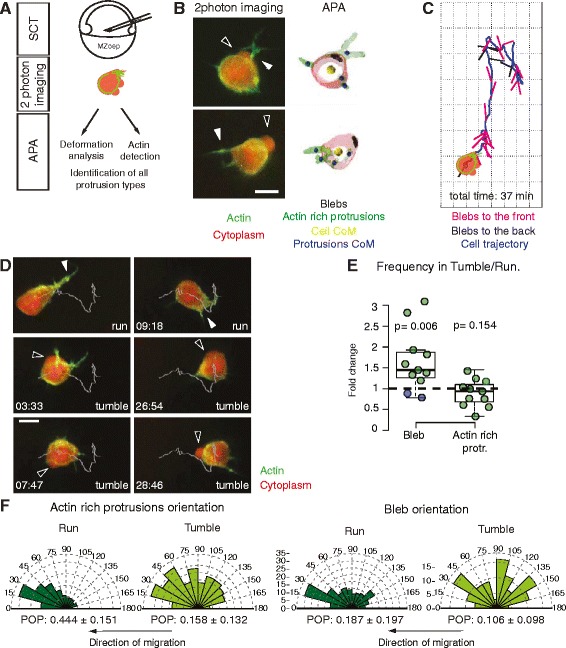
Analysis of protrusion orientation during single mesendoderm cell migration. **a** Cell migration and protrusion formation analysis procedure, from single mesendoderm cell transplantation to automatic protrusion analyzer (APA). **b** Left: Control cells displaying blebs (black arrowheads) and actin-rich protrusions (white arrowheads). Right: Corresponding cell outlines after APA processing, where the different protrusion types and the centers of mass (CoM) of cells and protrusions have been labeled. Scale bar = 10 μm. **c** Exemplary cell trajectory displaying unit vectors pointing from the cell CoM to the blebs CoM. Blebs are classified as forming towards the front if they form in the local direction of cell displacement. **d** Time lapse of a control mesendoderm cell transplanted in an MZ*oep* host displaying run and tumbles during migration. White line: trajectory of the CoM of the cell; white arrowheads: actin-rich protrusion; black arrowheads: blebs. Scale bar = 10 μm. Time in min:sec. **e** Frequency ratio of the formation of blebs and actin-rich protrusions during tumble versus run phases. The data points colored in blue correspond to cells where the reorientation events are associated with the formation of a new actin-rich protrusion at the leading edge. Note that the bleb frequency also includes the false negatives not detected by APA (Additional file 4: Figure S2). **f** Orientation of actin-rich protrusion and bleb formation in run and tumble phases. Arbitrary units (AU) are used for actin-rich protrusions as they are weighted with the total intensity of the Lifeact signal. The arrows below the diagrams indicate the local direction of cell migration. The overall orientation of each protrusion type was quantified using the polar order parameter (POP, see Additional file 1: Supplementary Methods for details). Mean ± SEM. In **b** and **d** cells express Lifeact-GFP (green) and Dextran-Alexa 594 (red). Number of cells in (**e, f**) = 11. Number of blebs in (**f**) = 349. Statistical significance by one-sided *t*-test (**e**) or by non-overlapping SEM of the POP (**f**) (Additional file 7: Figure S3D)

We have previously observed that enhancing bleb formation while reducing actin-rich protrusions in mesendoderm progenitors decreases the directional persistence of their migration [13]. We thus asked how the formation of different protrusion types relates to the run-and-tumbling behavior of mesendoderm progenitor cells. We acquired 10–30 min high-resolution two-photon microscopy movies of transplanted mesendoderm cells injected with Alexa594-Dextran to mark the cytoplasm and expressing Lifeact-GFP [18] to follow filamentous actin (Fig. 2a, b and Additional file 3: Movie 1). We observed that, similarly to collectively migrating prechordal plate cells [13], single mesendoderm progenitors formed blebs (spherical protrusions initially devoid of actin) and actin-rich protrusions (protrusions containing actin throughout their expansion) (Fig. 2b and Additional file 3: Movie 1).

To analyze the orientation of each protrusion type with respect to the direction of cell migration, we developed a new software package for three-dimensional (3D) cell and protrusion segmentation and automated detection and identification of individual protrusions (Automated Protrusion Analyzer (APA), Fig. 2a–c and Additional file 4: Figure S2). Protrusion identification and classification is based on detection of changes in cell surface curvature and morphological differences between protrusion types. APA identifies two types of protrusions: blebs and actin-rich protrusions (Fig. 2b). Actin-rich protrusions are distinguished from blebs by the presence of actin (labeled with Lifeact) in all phases of their expansion (Additional file 3: Movie 1), and by a higher curvature than blebs (Additional file 1: Supplementary Methods). Using APA, we could monitor the center of mass of the cells and each protrusion formed, as well as the intensity of actin in actin-rich protrusions during 3D migration (Fig. 2b, c). As lamellipodia size and actin content have been shown to correlate with migration speed [19], we analyzed the angle distribution of actin-rich protrusions weighted with the total intensity of the Lifeact signal in the protrusion. Thus, this weighted distribution mostly reflects the orientation of larger actin-rich protrusions. The overall orientation of a specific protrusion type was quantified using the polar order parameter (POP). The POP magnitude indicates how sharply focused the protrusion angle distribution is (Additional file 1: Supplementary Methods).

We then used these automated analysis tools to relate protrusion formation to mesendoderm progenitors’ run-and-tumbling behavior. Run-and-tumbling was evident in 11 out of 17 two-photon high-resolution timelapses (Fig. 2d); in the remaining timelapses, cells displayed directed motion only, likely because the shorter (10–30 min long) high-resolution movies necessary for protrusion analysis are sometimes too short to capture the tumbling behavior. Analysis of the timelapses where run-and-tumbling could be quantified showed that, during run phases, mesendoderm cells formed actin-rich protrusions in the direction of migration (Additional file 5: Movie 2, Fig. 2d–f) and poorly oriented blebs, as evidenced by the clear difference in POP between the two protrusion types (POP = 0.444 ± 0.151 for actin-rich protrusions vs. 0.187 ± 0.197 for blebs in run phases, mean ± standard error of the mean (SEM), Fig. 2f). In contrast, tumble phases were associated with the formation of an increased number of randomly oriented blebs (Fig. 2e) and a decrease in the focus of actin-rich protrusion formation (POP = 0.158 ± 0.132 for actin-rich protrusions formed during tumble phases, mean ± SEM, Additional file 5: Movie 2, Fig. 2f). In about 15 % of the tumble events, less blebbing was observed and a change in direction was achieved by the formation of a new leading edge actin-rich protrusion (corresponding to the two cells labeled as blue data points in Fig. 2e, Additional file 6: Movie 3). Taken together, our observations suggest that actin-rich protrusions may drive directed migration of mesendoderm progenitors whereas blebs mainly contribute to cell re-orientation.

### Modulating the proportion of blebs to actin-rich protrusions changes the ratio of tumbling to run times without affecting protrusion orientation

**Fig. 3:**
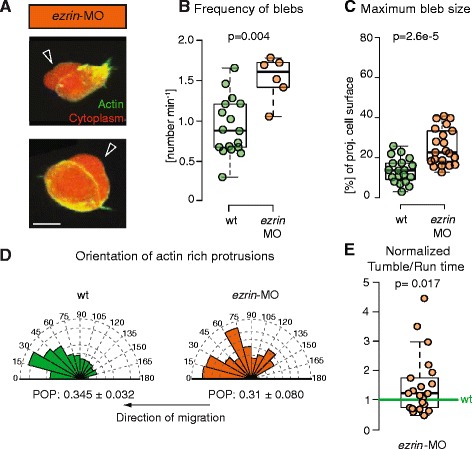
Protrusion formation and orientation in *ezrin* morphant mesendoderm cells. **a** Exemplary *ezrin*-MO-injected mesendoderm cells displaying blebs (black arrowheads). Cells express Lifeact-GFP (green) and Dextran-Alexa 594 (red). Scale bar = 10 μm. **b**, **c** Quantification of bleb formation frequency (**b**) and bleb size at maximal expansion normalized to cell size (**c**) in control and *ezrin*-MO-injected mesendoderm cells. Note that bleb frequency also includes the false negatives not detected by APA (Additional file 4: Figure S2). **d** Orientation of actin-rich protrusion formation in *ezrin*-MO-injected cells with respect to the local direction of migration. The arrows below the diagrams indicate the direction of migration. The orientation of actin-rich protrusions was weighted by their actin content (i.e., total Lifeact fluorescence) to account for size differences between protrusions, their number is thus given in arbitrary units. POP: mean ± SEM of the magnitude of the polar order parameter. **e** Ratio of tumbling to run times in migrating single lateral *ezrin* morphant mesendoderm cells (*ezrin*-MO). Cells were tracked during the approximately first 2 hours after transplantation. The ratio was normalized to transplanted control cells in the same embryo (internal controls) to account for experimental variability between different embryos. Number of analyzed cells in (**b**, **d**) = 17 for control and 6 for *ezrin*-MO; (**e**) = 21 for *ezrin*-MO. Number of blebs in (**c**) = 19 for control and 21 for *ezrin*-MO. Statistical significance by Mann–Whitney test (**b**, **c**), by non-overlapping SEM of the POP (**d**) (see also Additional file 7: Figure S3D) or by one-sided *t*-test (**e**)

To test whether the proportion of blebs to actin-rich protrusions formed by mesendoderm progenitors determines their run-and-tumbling behavior, we aimed to change the frequency of bleb formation. We increased bleb formation by reducing membrane-to-cortex attachment using a morpholino (MO) against *ezrin* [14], a protein that binds the actin cortex to the plasma membrane. Consistent with our previous observations in the prechordal plate [13], we found that single transplanted mesendoderm cells with reduced Ezrin activity showed a strong increase in the frequency and size of blebs and a reduction in actin-rich protrusions (Fig. 3a–c, Additional file 7: Figure S3A and Additional file 8: Movie 4). We previously showed that enhancing bleb formation by reducing Ezrin activity (either by expressing a dominant negative version of Ezrin or using a MO against ezrin) significantly reduces migration directional persistence, leading to less straight cell migration tracks in transplanted mesendoderm cells [13]. We thus asked whether the decrease in directional persistence in *ezrin*-MO cells was due to increased tumbling. Alternatively, reduced directional persistence could result from a change in the focus of protrusion expansion, as Ezrin depletion affects the entire cell and could affect overall cell polarity. To distinguish between these two possibilities, we analyzed protrusion orientation in *ezrin* morphant cells. We observed that the angle distributions of blebs and actin-rich protrusions were not affected by Ezrin depletion (Fig. 3d and Additional file 7: Figure S3B–D). We then analyzed the trajectories of transplanted progenitor cells during mid gastrulation stages (6–8 hpf) for control cells and *ezrin* morphant cells. We found that enhanced bleb formation in *ezrin* morphant mesendoderm progenitors significantly increased the ratio of the time spent tumbling to the time spent in run phases (Fig. 3e). This increase was due to a decrease in the duration of run phases (on average 5 min in control runs, *n* = 209, vs. 3.8 min in *ezrin*-MO runs, *n* = 231), while the duration of individual tumble phases was not significantly changed (on average 3.1 min in control tumbles, *n* = 216, vs. 3 min in *ezrin*-MO tumbles, *n* = 234).

**Fig. 4:**
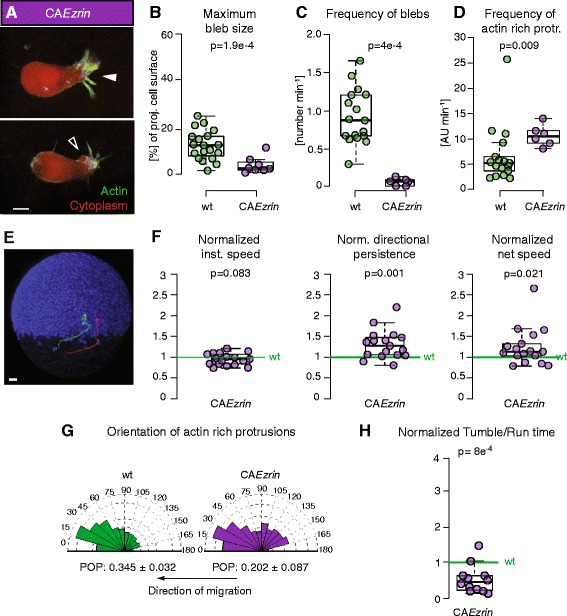
Protrusion formation and migration directionality in mesendoderm cells expressing CA*Ezrin*. **a** Exemplary actin-rich protrusion (white arrowhead) and bleb (black arrowhead) in CA*Ezrin-*expressing cells. Cells express Lifeact-GFP (green) and Dextran-Alexa 594 (red). Scale bar = 10 μm. **b**, **c** Quantification of bleb size at maximum expansion normalized to the cell size (**b**) and bleb formation frequency (**c**). Note that bleb frequency also includes the false negatives not detected by APA (Additional file 4: Figure S2). **d** Quantification of the frequency of formation of actin-rich protrusions. **e** Lateral view of a MZ*oep* mutant embryo (ectodermal nuclei are labeled with Histone-Alexa 647 in blue) at 60 % epiboly (7hpf) with example tracks of control (green) and CA*Ezrin*-expressing mesendoderm cells (red) transplanted into the lateral germ ring margin at 50 % epiboly (5.5 hpf). Tracking time = 110 min. Scale bar = 50 μm. **f** Ratio of instantaneous speed, directional persistence, and net speed of transplanted CA*Ezrin*-expressing single lateral mesendoderm cells. **g** Orientation of actin-rich protrusion formation in control and CA*Ez*rin cells. The arrows below the diagrams indicate the local direction of migration. POP: mean ± SEM. **h** Ratio of tumbling to run times in migrating single lateral mesendoderm cells expressing CA*Ezrin*. Cells were tracked during the approximately first 2 hours after transplantation. In f and h, values are ratio relative to transplanted control cells in the same embryo (internal controls) to account for experimental variability between different embryos (see also [13]). In d and g, arbitrary units (AU) are used as actin-rich protrusions weighted with the total intensity of the Lifeact signal in the protrusion. Number of blebs (b) = 19 for control and 8 for CA*Ezrin.* Number of cells in c, d, and g = 17 for control and 6 for CA*Ezrin*; (f) = 17 and (h) = 12 CA*Ezrin* compared to control. Statistical significance by Mann–Whitney test (b**–**d), one-sided *t*-test (f and h), or by non-overlapping SEM of the POP (**g**) (Additional file 7: Figure S3D)

We next sought to investigate how increasing the formation of actin-rich protrusions at the expense of blebs affects the run-and-tumbling behavior of mesendoderm progenitors. To this end, we increased membrane-to-cortex attachment by expressing a constitutively active version of Ezrin (CA*Ezrin*, T564D [20]). CA*Ezrin*-expressing transplanted single mesendoderm cells showed a strong decrease in blebbing activity and an increase in formation of actin-rich protrusions (Fig. 4a–d and Additional file 9: Movie 5). We then investigated how expression of CA*Ezrin* affected the migratory trajectories of single mesendoderm progenitors transplanted into MZ*oep* hosts from mid-to-late gastrulation stages (6–8 hpf). We observed that single CA*Ezrin* expressing mesendoderm progenitors showed an increase in migration directional persistence and net speed, while their instantaneous speed remained unchanged compared to co-transplanted control cells (Fig. 4e, f). We first checked whether this increase in directional persistence could result from an overall increase in the focus of protrusion formation upon expression of CA*Ezrin*. We found that the angle distribution of actin-rich protrusion formation was less focused in CA*Ezrin*-expressing cells than in control cells, indicating that the observed increase in cell directional persistence does not result from more focused actin-rich protrusions (Fig. 4g, Additional file 7: Figure S3C, D and Additional file 9: Movie 5). Bleb formation was rarely observed and only a few events could be analyzed (Fig. 4c and Additional file 7: Figure S3B). We then investigated whether expression of CA*Ezrin* affected the run-and-tumbling behavior of mesendoderm progenitors, and found that the ratio of tumbling to run times was decreased in progenitors expressing CA*Ezrin* (Fig. 4h). This decrease was due to an increase in the duration of run phases (on average 5 min in control runs, *n* = 209, vs. 6.4 min in CA*Ezrin* runs, *n* = 102), while the duration of individual tumble phases was not significantly affected (on average 3.1 min in control tumbles, *n* = 216, vs. 3 min in CA*Ezrin* tumbles, *n* = 104). Together, these observations suggest that the proportion of blebs to actin-rich protrusions controls the directional persistence of cell migration in mesendoderm progenitors by modulating the ratio of tumbling to run times.

### Modulating the ratio of tumbling to run times affects migration precision

**Fig. 5:**
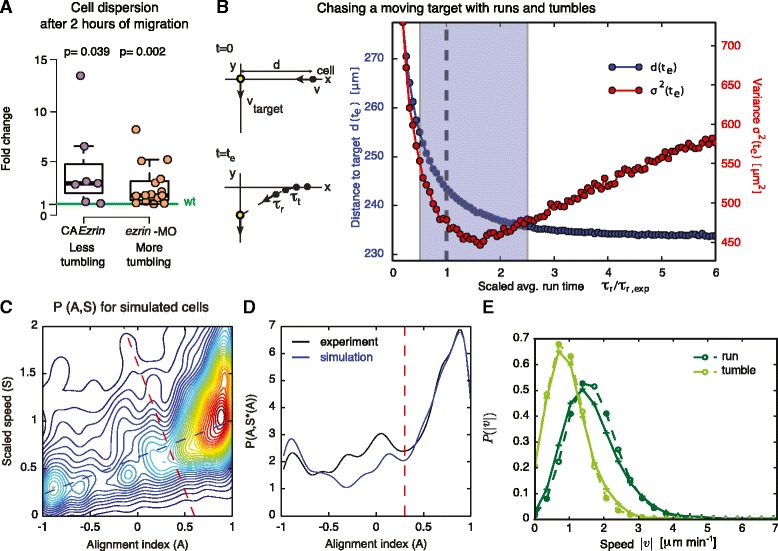
Modulating the ratio of tumbling to run times affects migration precision. **a** Positional variance of CA*Ezrin*-expressing and *ezrin*-MO cells after approximately 2 hours of migration. Values are the ratio relative to transplanted control cells in the same embryo (internal controls) to account for experimental variability between different embryos. **b** Schematic of chemotactic run-and-tumble migration: a cell (black) migrates towards a moving target (orange) via runs and tumbles of duration τ_r_ and τ_t_, respectively. After each tumble, the cell redirects towards the target. The target moves with a velocity *v*_*target,*_ and d is the initial cell-target distance. We evaluate the distance to the target after, t_e_ = 1.5 h. Simulation results for migration precision versus τ_r_/τ_r, exp_; τ_r_ is the run time in the model and τ_r, exp_ is the τ_r_ value extracted from fitting the model to experiments. Other parameters were chosen based on experimental measurements (Additional file 1: Supplementary Methods). Each point results from 100 simulations. Blue curve (d(*t*_*e*_)): mean target distance at time *t*_*e*_. Red curve: spatial dispersion of cells at *t*_*e*_. The blue shaded region corresponds to the range of τ_r,_ compatible with experimental observations (Additional file 10: Figure S4F, Additional file 1: Supplementary Methods). **c** Two-dimensional probability density of alignment and scaled speed, P(A,S), obtained from simulation of n = 23 model cells using parameters matching experimental data (Additional file 1: Table S1). The blue dashed line shows the linear fit to the maximum values of P(A,S) for A. The red dashed line is the line, perpendicular to the maximum, defining the threshold above which a portion of a trajectory is considered to be a run phase. **d** One-dimensional cross-section of P(A,S) along the maximum line from simulated cell trajectories in blue (**c**) and from experimental trajectories of controls transplanted into MZ*oep* hosts in black (data from Fig. 1h). Red dashed line as in c. **e** Speed distributions P(|v|) during runs and tumbles. Comparison of experimental controls transplanted into MZ*oep* hosts (crosses/solid lines) and model results (circles/dashed lines) for a single simulation run using parameters in Additional file 1: Table S1

Frequent direction changes have been proposed to enhance the precision of cell migration in complex environments, particularly during directed migration where the chemotactic target is moving or changing over time as might be the case during zebrafish gastrulation [9, 21]. Indeed, considering that mesendoderm cells migrate dorsally and vegetally towards the forming body axis, it is commonly believed that they follow a chemotactic signal from the epiboly front. We thus asked whether changing directional persistence affects the overall precision of mesendoderm progenitor migration. We assessed the precision of cell migration by quantifying the spatial dispersion after approximately 2 hours of migration of cells that were co-transplanted at the same location at 50 % epiboly, for cells with different levels of Ezrin activity. Interestingly, we found that both the cells displaying enhanced blebbing and tumbling, and the cells displaying enhanced formation of actin-rich protrusions and running, had a significantly higher spatial dispersion than control cells (Fig. 5a). These observations suggest that both decreasing and increasing the ratio of tumbling to run times in mesendoderm progenitors decreases the precision of cell migration.

To test whether the ratio of tumbling to run times observed in mesendoderm progenitors might indeed optimize migration precision, we developed a stochastic model of cells migrating towards a target moving at constant speed. We represented the moving cells by active Brownian particles randomly switching between run and tumble phases (Fig. 5b, Additional file 1: Supplementary Methods, Additional file 10: Figure S4 and Additional ﻿file 11: Figure S5). During run phases cells perform directed active Brownian motion with stochastic speed and a direction fluctuating around a mean value oriented towards the target with a detection error. During tumble phases cells are randomly moving without any preferred direction. We constrained the model parameters by comparing characteristic observables of motion obtained from simulated tracks (analyzed with the same procedure as applied to the experimental data) to experimental measurements. Specifically, several parameters describing cell velocity, as well as run and tumble durations were compared between simulations and experiments. A parameter search yielded a set of parameters very accurately accounting for measured experimental values in control mesendodermal cells (Additional file 1: Table S2 and Additional file 1: Supplementary Methods for details). We found that, with this selected set of parameters, the combined 2D distribution of alignment and cell speed, and the probability distribution of cell speeds during run and tumble phases were well captured by the simulations without further fitting (Fig. 5c, compare to Fig. 1g, and Fig. 5d, e). These observations indicate that the numerical model accurately captures the aspects of cell migration relevant to the observed progenitor trajectories.

Using the estimated parameters, we then systematically varied the run time of the model cells and assessed the precision of cell migration by computing the distance to target and the dispersion of the cell population at the end of the experiment (t_e_ = 1.5 h). We found that the distance to target decreased as a function of the run time, indicating that longer runs are more favorable for overall cell velocity. Strikingly, cell dispersion showed a clear minimum around the mean run time measured for control mesendoderm progenitors. This prediction is consistent with the increased cell dispersion measured for CA*Ezrin* and *ezrin*-MO cells (Fig. 5a), which display run times longer and shorter than control cells, respectively. Taken together, our experiments and model thus indicate that the ratio of tumbling to run times is a critical factor controlling the precision of cell migration in vivo.

## Discussion

Low directional persistence is often thought to be a consequence of a shallow chemotactic gradient resulting in the formation of unfocused protrusions [1, 3]. Here, we show that the directional persistence of zebrafish mesendoderm progenitors migrating in vivo does not depend on the directional focus of protrusion formation, but rather is determined by the ratio of persistent run phases to tumble phases associated with cell reorientation. Interestingly, progenitor cells appear to control the ratio of tumbling to run times by adjusting the proportion of blebs to actin-rich protrusions formed during migration. Blebs have previously been implicated in mediating directed migration of primordial germ cells during zebrafish embryogenesis [22], and of a number of cancer lines in culture and in vivo [23, 24]. In zebrafish primordial germ cells, bleb growth appears to expand the cell body forward, and subsequent anchoring of the bleb neck to the substrate by adhesive contacts to surrounding cells is thought to drive cell migration [25]. Our finding that blebs in mesendoderm progenitor cells are predominantly associated with tumbling reorientation events suggests that, in these cells, blebs are primarily used for exploring the environment, whereas actin-rich protrusions drive directed migration during run phases. Specifically, undirected bleb formation, as observed during tumble phases, induces displacement of the cell towards random directions and might thus provide a stochastic way of exploring the environment. This difference in bleb function between primordial germ cells and mesendoderm cells may be due to the fact that mesendoderm progenitors form directed actin-rich protrusions, whereas primordial germ cell migration appears to rely exclusively on blebs [9].

The run and tumbling behavior of control mesendoderm progenitors appears highly similar for cells in wt and in MZ*oep* hosts. Furthermore, our experiments indicate that the ratio of run and tumbling can be modulated in single transplanted cells by tuning the amount of Ezrin activity. To account for experimental variability between different embryos, cells with increased or decreased Ezrin activity were always co-transplanted with control cells in the same MZ*oep* embryo (internal controls) (see also [13]). These observations indicate that run and tumbling is largely a cell autonomous behavior. Nonetheless, it remains to be investigated whether extracellular factors, such as the distribution, organization and nature of extracellular matrix or the proximity to the chemotactic signal followed by the cells, influence run and/or tumbling in zebrafish mesendoderm progenitors.

Run-and-tumbling is a common feature of bacterial chemotaxis, where it is a strategy for efficient gradient sensing [26], but has also been observed in a variety of eukaryotic motile cells, including primordial germ cells [9], chlamydomonas [27], and mammary epithelial cells [28]. Bacteria are too small to accurately measure a chemoattractant gradient without moving, and use temporal comparisons instead, leading to a biased random walk with longer run phases in the direction of the chemotactic gradient. Animal cells are large enough to polarize in a gradient without motion [29] and thus alternating run and tumbling phases during migration is likely to serve a different function than in bacterial chemotaxis. It has been speculated that tumble-associated direction changes might increase the precision of chemotactic cell migration in animal cells [12, 21]. Our observation that changing the ratio of tumbling to run times impairs the focus of cell migration provides direct experimental evidence supporting this hypothesis. Indeed, both increasing and decreasing the tumbling to run ratio by modulating the bleb-to-actin-rich protrusion ratio led to impaired cell migration precision (Fig. 5a). Distinct molecular pathways regulate the formation of blebs and actin-rich protrusions [23, 30], suggesting that the ratio between the two protrusion types could be readily tuned. Such a sub-specialization of protrusion function would allow cells to easily modulate the frequency of re-orientation events during migration in complex and changing environments. Our theoretical model, which recapitulated key features of mesendoderm progenitor migration, predicts that an optimal tumble to run ratio enhances migration precision. Indeed, too long runs increase cell dispersion by overly amplifying initial errors in migration direction, whereas too short runs increases cell dispersion because frequent direction changes enhance heterogeneity in direction between cells. Furthermore, it is possible that alternating run and tumbles enhances the robustness of migration to noise in, for example, lamellipodium orientation [31].

## Conclusions

Our experiments and model indicate that mesendoderm progenitors may be operating close to an optimum tumbling to run ratio for precise migration in the in vivo context of the developing zebrafish embryo. Taken together, our data suggest that, by adjusting the proportion of blebs to actin-rich protrusions, mesendoderm cells modulate the ratio of run to tumbling times, and thereby control the precision of their migration. A number of cell types have been reported to combine blebs and actin-rich protrusions during migration [32–35]. Future studies will have to investigate whether blebs and actin-rich protrusions also have distinct functions in these cells types.

## Methods

### Embryo staging and maintenance

Zebrafish maintenance was carried out as described [36]. Embryos were grown at 31 °C in E3 medium and staged as described previously [37].

### MRNA, morpholino, and dye injection

mRNA was synthesized as previously described [38]. For single cell transplantation, wt TL embryos were injected with 50 pg of Lifeact-GFP [18], 3.25 ng of Dextran Alexa Fluor-595 (D22913, Invitrogen), and 100 pg of cyc alone (control) or together with 4 ng of *ezrin*-UTR-MO [14], to generate *ezrin*-MO cells or 150 pg of CA*Ezrin* mRNA (T564D of *Danio rerio*’s gene as in [20]) to generate CA*Ezrin* cells.

For tracking of cell nuclei in low magnification transplantation experiments, wt donor embryos were injected with 100 pg of cyc together with Alexa Fluor-488 conjugated histone H1 (H13188, Invitrogen) (control), or 100 pg of histoneH2Azf::mcherry plus 150 pg of CA*Ezrin* mRNA (CA*Ezrin* cells). MZ*oep* host embryos were injected with Dextran Alexa Fluor-647 (D22914, Invitrogen) (see also [13]).

### Transplantation experiments, cell imaging, and bleb size measurements

For transplantation experiments, wt and experimental TL donors and MZ*oep* dharma::GFP host embryos were dechorionated with Pronase (2 mg/mL in E2) and transferred onto an agarose plate with E3 medium. Two to three cells were taken from control and experimental donor embryos at dome stage (4.5 hpf) and co-transplanted into the emerging lateral mesendoderm of a host embryo labeled with Dextran Alexa Fluor-647 at 50 % epiboly (5.5 hpf).

For low magnification experiments, time-lapse images were obtained with an upright Leica SP5 confocal microscope equipped with a 20× water immersion lens, using 488-nm Argon, DPSS 561 nm, and 633-nm HeNe laser lines. Frames were captured at 90 s intervals for 3 h (~5.5–8.5 hpf). The temperature was kept constant in all videos (28 °C).

For big magnification transplantation experiments, images were obtained with a Zeiss 710 two-photon microscope equipped with a 63×/1.2 objective, using 910 nm wavelength of the Chamaleon laser. Frames were captured at 10–25 s intervals for 10–30 min, between 6 and 8 hpf.

For bleb size measurements, the projected area of each bleb at its maximal extension was measured using ImageJ and normalized to the projected area of the whole cell.

For cell dispersion measurements, pictures were taken with a dissecting microscope (Olympus SZX 12) equipped with a QImaging Micropublisher 5.0 camera approximately 2 hours post-transplantation.

### Image analysis

For single transplanted cells in low magnification movies nuclei tracking in three dimensions (x, y, and z) was performed with Imaris 7.3.0 software. The instantaneous and net speeds, as well as directional persistence (ratio of the net displacement to the distance actually traveled by the cells), were extracted from the tracks.

Analysis of the directions of protrusion formation in combination with cell tracking in higher magnification movies was performed using the APA software, described in Additional file 1: Supplementary Methods.

### Statistical analysis

*t* tests were performed after the data were confirmed to have normal distribution and equal variance; otherwise, Mann–Whitney U tests were applied. *P* values were computed in R. For low magnification cell transplantation experiments and variance of cell position (used to assess cell dispersion), one-sided *t* test was used, which compared experimental data points to an equal sized group of 1. We also computed the *P* values with ttest2 from Matlab, which compared experimental data points with a random distribution of numbers around one with the same standard deviation as our data. ttest2 yielded similar results and conclusions.

To numerically describe the angular distribution of protrusions, we used the polar order parameter (POP), as explained in detail in Additional file 1: Supplementary Methods. We consider two POP values to be significantly different when their SEMs do not overlap.

### Definition of run-and-tumbling phases

For longer trajectories (Figs. 1 and 4e, f), a timeframe of 1.5 min was used as it maximized the amount of embryos we could image simultaneously without a change in the run-to-tumble behavior or in the instantaneous speed. Run-and-tumbling phases were automatically extracted using and unbiased procedure described in Additional file 1: Supplementary Methods [39, 40]. For the analysis of short cell trajectories (timeframe ~10 s, Figs. 2 d–f, 3e, 4 h), “runs” were defined as phases where the trajectory does not deviate by more than 45 degrees from the direction at the beginning of the run or if a change in direction larger than 45 degrees persists for less than 5 timeframes. “Tumbles” were defined as phases where a change of direction higher than 45 degrees occurs and persists for longer than 5 timeframes.

### Measurements of cell dispersion

Cell dispersion was assessed using cell position variance, as measured by adding the variances in x and y of the positions of control and experimental cells approximately 2 hours after they were co-transplanted at the same location in a host embryo at 50 % epiboly. Only embryos with at least three control cells and three experimental cells were considered. The ratio has been normalized to transplanted control cells in the same embryo (internal controls) to account for experimental variability between individual transplantation experiments.

## Additional files

Additional file 1:
**Supplementary Methods.** Supplementary methods file with detailed description of: 1. Data analysis: (A) Automatic Protrusion Analyzer (APA) software. (B) Polar Order Parameter (POP) used to characterize the distribution of orientations of protrusions of the model. (C) Automatic detection of run and tumbles. 2. Model of cell migration: (A) Cell migration model description [41–44]. (B) Parameterization of the computational model using experimental measurements [45]. (C) Model Predictions – Distance to target and position variance [31]. (PDF 203 kb) Additional file 2: Figure S1. Durations of run and tumble phases. Distribution of run and tumble durations as detected by our algorithm from low-magnification movies of wt cells transplanted in a MZ*oep* host. Frames were captured at 90 s intervals for 3 h (~5.5–8.5 hpf). The inset shows the same data in a semi-logarithmic plot, where a line indicates an exponential relationship. Exponentially distributed times are expected in a run and tumble trajectory if we assume a simple two-state Markov processes, namely a process that undergoes transitions from one state to another where the probability distribution of the next state depends only on the current state and not on the sequence of events that preceded it [41]. (PDF 103 kb) Additional file 3: Movie 1. Protrusion formation in a single wt transplanted mesendodermal cell. Time-lapse of a wt mesendoderm progenitor (left) and the same cell segmented using APA (right). See also Fig. 1b. Plasma membrane (GPI-RFP) is red; actin cortex (Lifeact-GFP) is green. Scale bar = 10 μm. Time in minutes:seconds. (MP4 21029 kb) Additional file 4: Figure S2. Accuracy of protrusion detection using the Automatic Protrusion Analyzer (APA). APA accurately detects 83 % of the blebs formed by the cells as validated by two independent experimentalists. The majority of false positive detected blebs consisted of the same bleb being detected at more than one time point. Number of analyzed cells = 28. Manual control was also performed for actin-rich protrusions and detection was found to be accurate for more than 99 % of the protrusions and for this reason we do not provide the results of the manual segmentation here. (PDF 148 kb) Additional file 5: Movie 2. Run and tumbling during migration of a control mesendoderm progenitor. Example time-lapse of a transplanted mesendoderm control progenitor alternating phases of directed migration (run) and phases when the cell temporarily stalls, preceding reorientation (tumbles). Plasma membrane (GPI-RFP) is red; actin cortex (Lifeact-GFP) is green. Scale bar = 10 μm. Time in minutes:seconds. (MP4 14122 kb) Additional file 6: Movie 3. Cell reorientation by formation of a new actin-rich protrusion. Example time-lapse of a transplanted mesendoderm control progenitor changing migration direction by forming a new leading edge actin-rich protrusion. Plasma membrane (GPI-RFP) is red; actin cortex (Lifeact-GFP) is green. Scale bar = 10 μm. Time in minutes:seconds. (MP4 11663 kb) Additional file 7: Figure S3. Frequency of actin-rich protrusions, orientation of cell protrusions, and summary of POP values. (A) Frequency of actin-rich protrusions in control and *ezrin*-MO-injected mesendoderm cells. Arbitrary units (AU) are used as actin-rich protrusions are weighted with the intensity of the Lifeact signal in the protrusion. (B) Orientation of blebs with respect to the local migration axis. POP: Mean ± SEM of the magnitude of the polar order parameter. Number of analyzed cells = 17 for wt, 6 for *ezrin*-MO, and 6 for CA*Ezrin*. Number of blebs n = 349 for wt, 163 for *ezrin*-MO, and 6 for CA*Ezrin*. Statistical significance was determined comparing the mean ± SEM of the magnitude of the POP of the angular distributions. (C) Orientation of bleb and actin-rich protrusion formation with respect to the Yolk-ectoderm axis. For all experimental conditions, protrusions are almost exclusively oriented perpendicular to the Yolk-ectoderm axis, indicating that protrusions are formed into the extracellular space between the Yolk cell and the overlaying ectoderm layer. (D) Mean values ± SEM of the magnitude of the POP of the angular distributions of the analyzed protrusions. Green shaded area covers the wt mean ± SEM. Number of analyzed cells = 17 for wt, 6 for *ezrin*-MO, and 6 for C*AEzrin*. Number of actin-rich protrusions = 10853 for wt, 1501 for *ezrin*-MO, 1160 and 2549 for CA*Ezrin*. Number of blebs n = 349 for wt and 163 for *ezrin*-MO. For CA*Ezrin* only 6 blebs were observed so the POP was not calculated. (PDF 600 kb) Additional file 8: Movie 4. Bleb formation is enhanced in ezrin morphant single transplanted mesendodermal cells. Example time-lapse of an *ezrin*-MO injected transplanted mesendoderm progenitor. Plasma membrane (GPI-RFP) is red; actin cortex (Lifeact-GFP) is green. Scale bar = 10 μm. Time in minutes:seconds. (MP4 30553 kb) Additional file 9: Movie 5. Formation of actin-rich protrusions is enhanced in CA*Ezrin*-expressing single transplanted mesendodermal cells. Example time-lapse of a transplanted mesendoderm progenitor expressing CA*Ezrin*. Plasma membrane (GPI-RFP) is red; actin cortex (Lifeact-GFP) is green. Scale bar = 10 μm. Time in minutes:seconds. (MP4 43410 kb) Additional file 10: Figure S4. Computational model description and supplementary results. (A) Schematic visualization of the simulation set-up: three-dimensional model where the cell is confined in the z-direction to the mesoendodermal layer of height Δ_ME_. The red arrow indicates the direction of polarization within the layer. (B) Schematic visualization of example model trajectories in the mesoendodermal plane (xy) in the tumble and run phases. The arrow in the run panel indicates the direction of the run determined by the polar angle 'φ_D_ = 0, which sets the average direction of migration during the run. The dashed lines indicate the instantaneous movement angle 'φ(t_0_) at time t_0_. Impact of orientation error ε on the model results (C–E). Distance to target and spatial variance of the cells at t = 90 min as a function of the scaled avg. run time τ_r_ for different values of the reorientation error towards the target ε (value estimated for wt cells and used in the main text ε = 0:2, Fig. 5); (C) ε = 0:1, (D) ε = 0:3, and (E) ε = 0:4. Average relative error versus run time (F). Average relative error *E*_*rel*_ defined as the mean observable deviation (Additional file 1: Table S2) between model simulations and experimental results (wt cells in MZ*oep* host). All model parameters as fitted to experimental data with the average run time being varied around the fitted value as in Fig. 5b. The vertical line represents the threshold value 0.1, which defines the shaded ‘consistency region’ in Fig. 5b. (PDF 1475 kb) Additional file 11: Figure S5. Comparison of experimental and simulated cell trajectories (A). Examples of cell trajectories from wild-type cells in an MZ*oep* host from low-magnification experiments (top) and from model simulations with the fitted parameters (bottom). All experimental (three-dimensional) trajectories were projected to the primary two-dimensional plane of their motion and rotated so that their average direction of migration aligns with the y-axis as in the simulations. Frames were captured at 90 s intervals for 3 h (~5.5–8.5 hpf). The red symbols indicate the points along the trajectory identified as tumbling events using the algorithm introduced in Sec. I C. Simulated cell trajectories corresponding to different experimental conditions (B). Examples of simulated cell trajectories for different values of model parameter τ_r_ with other parameters as fitted for wt cells in an MZ*oep* embryo. (left) τ_r_ as fitted to the wt cells (τ_r_ = 8 min). (center) For long runs (τ_r_ = 25 min), which yield *T*_*r*_ *= T*_*t*_ - values consistent with results obtained from CA*Ezrin* cells, and (right) short runs (τ_r_ = 4 min), which yield *T*_*r*_ *= T*_*t*_, values consistent with the ones measured for *ezrin*-MO cells. (PDF 1651 kb)

## Abbreviations

Cyc: Nodal-ligand cyclops
wt: Wild type
hpf: Hours post-fertilization
SD: Standard deviation
S: Scaled speed
A: Alignment index (a measure of the local persistence)
MZ*oep*: Maternal zygotic oep
GFP: Green fluorescent protein
APA: Automated protrusion analyzer
POP: Polar order parameter
SEM: Standard error of the mean
MO: Morpholino
CA*Ezrin*: Constitutively active version of Ezrin
