# Supplementary material for: Steering cell migration by alternating blebs and actin-rich protrusions
Source: BMC Biol. 2016 Sep 2;14(1):74. doi: 10.1186/s12915-016-0294-x (PMC5010735; doi:10.1186/s12915-016-0294-x)
Supplement: Additional file 1: — Supplementary Methods. Supplementary methods file with detailed description of: 1. Data analysis: (A) Automatic Protrusion Analyzer (APA) software. (B) Polar Order Parameter (POP) used to characterize the distribution of orientations of protrusions of the model. (C) Automatic detection of run and tumbles. 2. Model of cell migration: (A) Cell migration model description [41–44]. (B) Parameterization of the computational model using experimental measurements [45]. (C) Model Predictions – Distance to target and position variance [31]. (PDF 203 kb) [file 12915_2016_294_MOESM1_ESM.pdf]

SUPPLEMENTARY METHODS

Steering cell migration by alternating blebs and actin-rich protrusions

Alba Diz-Muñoz, Pawel Romanczuk, Wemiao Yu, Martin Bergert, Kenzo Ivanovitch, Guillaume Salbreux, Carl-Philipp Heisenberg and Ewa Paluch

I. DATA ANALYSIS

A. Automatic Protrusion Analyzer (APA) software: Automatic cell segmentation, detection of actin rich protrusions and detection of blebs

The Automated Protrusion Analyzer (APA) requires 3 pre-processing steps.

- A normalization of the voxel size (the z resolution in our experiments is always 1.0 μm, while the resolution of x and y varies). The image stacks are reconstructed using a linear interpolation such that a voxel in the reconstructed image stack has the same x, y and z resolution (0.25 μm).
- Smoothing with a Gaussian kernel to reduce the noise in the reconstructed image.
- Since there might be more than one cells in the field of view, a marker is manually created at the first time point to highlight the cell of interest that will be then tracked at different time points.

Cell segmentation and identification of protrusions are then achieved in the following 3 steps:

1. **Cell segmentation:** The cell is segmented based on a combination of independent segmentations of the RFP and GFP channels. For the RFP channel, a threshold for the cytoplasmic signal (Alexa594-Dextran) channel is automatically determined at the first time point to separate the cell from the background. We assume that the cell volume does not substantially change during migration; we thus optimize the threshold value at each time point such that the cell volume is constrained. Segmentation in the GFP channel (Lifeact-GFP) is performed similarly to the processing of cytoplasmic signal. The only difference is that subtle structures, such as fine and long protrusions are enhanced before the segmentation.
2. **Identification and classification of different structures in a cell:** At each timepoint, APA identifies and classifies 3 cellular structures: the cell body, actin rich protrusions and blebs. The identification of the cell body and actin rich protrusions use a morphology operation opening, i.e. dilation followed by erosion. Given a ball element structure for the opening operation, fine and long structures are removed. We classify the bulky volume as cell body. The rest of the cell detected in the GFP channel (Lifeact-GFP) is classified as actin rich protrusions. Blebs are identified as spherical deformations of the cell body that occur faster than the average cell movement and that do not overlap with an actin rich

protrusion. To accurately detect the blebs, we analyze the dynamics of cell deformations. For a given cell segmentation at two continuous time points, cell deformation is calculated based on minimum distance mapping. A bleb is then identified as a bigger deformation in a particular surface area.

3. **Extraction of positional information**: The cell center of mass is defined as the geometrical center of the entire segmented cell and is tracked over time. Additionally, APA tracks the geometric center of blebs, and the center of mass, the volume and the overall brightness (sum of the brightness of each voxel in a given protrusion) of actin rich protrusions.

In order to test the accuracy of this automatic segmentation and to avoid false positives two independent experimentalists manually checked every automated protrusions detection. 83% of the blebs and over 99% of the actin rich protrusions observed by the experimentalists were successfully detected by APA. False positives are usually blebs detected in more than one time frame, they were removed from the output of the APA analysis. About 17% of the blebs formed by the cell were not detected by the software (Fig. S2), these were manually added to the data for further processing.

B. Polar Order Parameter (POP)

In order to quantitatively analyze the orientation of blebs and actin-rich protrusions, we computed a polar order parameter (POP) vector $\mathbf{p}$ characterizing the distribution of orientations of protrusions. The orientation of the POP vector corresponds to the mean orientation of the protrusions and its magnitude characterizes how sharply peaked the angle distribution is. To evaluate the POP of a cell population, we first evaluated the order parameter $\mathbf{p}^i$ for each cell, with $i = 1...N$ index the individual cells:

$$p_x^i = \frac{\sum_{k_i} I(k_i) \cos\left(\phi(k_i)\right)}{\sum_{k_i} I(k_i)} \tag{1}$$

$$p_y^i = \frac{\sum_{k_i} I(k_i) \sin\left(\phi(k_i)\right)}{\sum_{k_i} I(k_i)} \tag{2}$$

where $k_i = 1...N_i$ index the successive protrusions formed by the cell i, $I(k_i)$ is the intensity of protrusion k_i and $\phi(k_i)$ is the angle of protrusion k_i. The intensity of a protrusion $I(k_i)$ is defined as being 1 for blebs, and as the total intensity of the protrusion for actin-rich protrusions.

The total POP of a given cell population was then obtained by averaging the order parameter in different cells:

$$p_x = \langle p_x^i \rangle, \qquad p_y = \langle p_y^i \rangle \tag{3}$$

where $\langle \cdot \rangle$ represents a weighted averaging operator taking into account the total intensity of protrusions formed by each cell:

$$\langle X \rangle = \frac{1}{\sum_{\text{cell i}} \sum_{k_i} I(k_i)} \sum_{\text{cell i}} \left(\sum_{k_i} I(k_i) \right) X_i \tag{4}$$

Because individual protrusions can not be resolved, we weighted the orientation of protrusions by their measured intensity in the averaging of Eq. 4, assuming that different protrusions contribute additively to the total measured intensity. The standard deviation σ of the POP for a given cell population was then calculated using:

$$\sigma_x^2 = \langle (p_x^i)^2 \rangle - \langle p_x^i \rangle^2 \tag{5}$$

The standard error of the mean on the POP of a cell population was then defined as SEM $= \sigma/\sqrt{n}$, where n is the total number of cells analyzed. POP values of distinct cell populations were considered significantly different when their respective POP$\pm$SEM did not overlap.

C. Trajectory analysis and automated detection of run and tumbles

In this section, we describe the automated procedure used to analyse cell trajectories and separate them into run and tumble phases. We introduce two observables of cell motion, the alignment index A, measuring the local persistence of cell motion, and the normalized cell speed S. High values of the alignment index and cell speed are associated with run phases where the cell performs directed motion, and low values with tumble phases. By considering the values of (A, S) obtained from cell trajectories, we define an objective criterion allowing us to separate cell trajectories into regions of low and high persistence and speed, corresponding to tumble and run phases, respectively.

Cell trajectories are characterized by a set of positions $\mathbf{r}(t_i)$, obtained at successive observation times t_i. The velocity vector $\mathbf{v}(t_i)$ is determined by calculating the differences of the position vectors $\mathbf{r}(t_i)$ at subsequent times, and dividing by the time interval Δt:

$$\mathbf{v}(t_i) = \frac{1}{\Delta t} \left(\mathbf{r}(t_i) - \mathbf{r}(t_{i-1}) \right) \tag{6}$$

From the velocity vectors, we define the alignment index A as a measure of the local persistence of the cell trajectory a time t_i:

$$A(t_i) = \frac{\mathbf{v}(t_{i+1}) \cdot \mathbf{v}(t_i)}{|\mathbf{v}(t_{i+1})||\mathbf{v}(t_i)|}, \tag{7}$$

$A(t_i)$ is a scalar which takes values between -1 and $+1$. $A(t_i) = 1$ corresponds to perfectly straight motion of the cell, $A(t_i) = -1$ indicates a complete reversal of the cell direction (turn by $180°$), whereas $A(t_i) = 0$ corresponds to a turn by $90°$.

In addition to the alignment index, we use the velocity vectors to define the normalized speed S:

$$S(t_i) = |\mathbf{v}(t_i)|/\langle|\mathbf{v}|\rangle \tag{8}$$

where $|\mathbf{v}(t_i)|$ is the instantaneous speed, and $\langle|\mathbf{v}|\rangle$ is the average speed of the cell over its entire trajectory. The normalization by the speed averaged over the trajectory is performed because different cells may exhibit significantly different average speeds of migration. S therefore yields a local estimate of whether a cell is moving slower or faster than its mean speed, with $S(t_i) = 1$ corresponding to a cell moving at its mean speed.

We then obtain a time series $(A(t_i), S(t_i))$ for each cell. The time series is smoothed using a moving average filter with the Hamming function with window size W [39]. The smoothing of the time series limits the impact of large deviations in the data due to detection noise. We verified that although different averaging windows and functions may affect the quantitative values of the detected run and tumble times $\tau_{r/t}$, the overall qualitative results remain unchanged by this choice. It was ensured that comparisons between experimental data and model results was performed using the same moving smoothing procedure. All presented results were obtained using $W = 3$ (smallest possible window size).

The resulting smoothed time series $(A(t_i), S(t_i))$ from all cell trajectories ($N = 23$) were then combined to yield estimate a two dimensional probability density $P(A, S)$ (Figure 1C,G and Figure 5C). This procedure implicitly assumes stationary migration dynamics, so that values of A and S at different times arise from the same probability density. The continuous probability density $P(A, S)$ was obtained from the discrete set of values of $(A(t_i), S(t_i))$ by using a Gaussian kernel estimation method. The bandwidth parameters were selected based on the corresponding data sets using Scott's rule [40].

For data obtained from wild-type cells transplanted into a wildtype or MZ*oep* host, the resulting probability density appears multi-modal, with several maxima (Figure 1C,D,G,H and Figure 5C,D).

We noticed that the positions of the maxima of $P(A, S)$ are approximately located on a straight line in the space (A, S). By performing a linear fit to the position of the maximum values of $P(A, S)$ as a function of A, we obtain a linear approximation of the *maximum line* (see Fig. 1C,D,G,H & 5C,D). The positive slope of the *maximum line* reflects the positive correlation between the normalized speed S and the alignment index A (Spearman $\rho = 0.67$, $r = 1 \cdot 10^{-7}$), with higher probability to observe faster cell migration together with more directed migration. We use the structure of $P(A, S)$ for automatic classification of run and tumbles, by calculating a *threshold line* (red dashed line in Fig. 1C,D,G,H & 5C,D) perpendicular to the *maximum line*, intersecting it at $A = 0.52$ for the wild-type host and at $A = 0.3$ for the MZ*oep* host. This alignment values correspond to the minimum between the first (global) and the second maximum.

Because of the low number of observations for $A \leq 0$ the detailed structure of the $P(A, S)$ at low A (number and positions of small relative maxima) is not reliable and depends most likely on the experimental realization. This however does not affect the position of the threshold line.

We then use the *threshold line* for automated detection of run and tumbles states along each individual trajectory. Points $(A(t_i), S(t_i))$ belonging to the region on the left of the threshold line (corresponding to small alignment index A) are associated with tumbles, and conversely points belonging to the right region are associated with runs. Thus, "runs" correspond to sequences of trajectory with fast, persistent migration, interrupted by phases with slow and non-directional migration.

Based on this classification, trajectories can be divided into sequences of runs and tumbles in an unbiased manner. The times spent in each phase yield the average run and tumble times T_r and T_t. We also introduce 3 additional observables conditioned on the trajectory state (run versus tumble):

1. average absolute value of mean velocity during run/tumble:

$$\langle|\bar{\mathbf{v}}|\rangle_{\mathrm{t/r}} = \langle\frac{1}{2}\,|\mathbf{v}(t_{i+1}) + \mathbf{v}(t_i)|\rangle_{\mathrm{t/r}},$$

2. average absolute value of velocity deviations during run/tumble:

$$\langle|\Delta\mathbf{v}|\rangle_{\mathrm{t/r}} = \langle\frac{1}{2}\,|\mathbf{v}(t_{i+1}) - \mathbf{v}(t_i)|\rangle_{\mathrm{t/r}},$$

3. average speed during run/tumble:

$$\langle\overline{|\mathbf{v}|}\rangle_{\mathrm{t/r}} = \langle\frac{1}{2}\,(|\mathbf{v}(t_{i+1})| + |\mathbf{v}(t_i)|)\rangle_{\mathrm{t/r}},$$

These observables yield informations on the properties of cell migration trajectories in the run and tumble phases. For the average absolute mean velocity (1) and average speed (3), a moving average with two points was chosen, ensuring that these quantities are calculated from the same velocities as the average velocity deviations, (2).

Overall, 8 observables (the average experimentally measured run and tumble times T_r and T_t, and the quantities (1)-(3) for runs and tumbles) obtained from the wild-type cells in the MZ*oep* host were used to parametrize the computational model, as described in the next section.

II. MODEL OF CELL MIGRATION

We describe here a stochastic model of cell migration which captures the essential features of the migration trajectories of mesendodermal cells. The migrating cell is represented by a particle moving in space. The particle switches between a tumble state, where it performs a random motion with no directionality, and a run state, where the particle moves towards a target with a noisy speed and direction (section II A). We use a fitting procedure to determine the parameters of the model producing trajectories which are statistically similar to recorded wild-type cells trajectories (section II B). We find an excellent agreement between the model and experimental data (Fig. 5C-E). We then use the model to explore the effect of varying the ratio of the times spent in run and tumble phase (section II C). We find that an optimal run to tumble ratio exists which minimises the dispersion of a group of moving cells.

A. Cell migration model description

We model individual cells as stochastic agents moving in a three dimensional space. Measured experimental trajectories indicate that cells are confined in a thin layer parallel to the surface of the embryo. The center of mass of the agents $\mathbf{r}(t)$ is therefore assumed to be confined in z-dimension with $-\Delta_{ME}/2 < z < \Delta_{ME}/2$, such that the agents move in a thin mesendodermal 2d-layer of the thickness Δ_{ME} (see Fig. S4). Because the agent position corresponds to the cell center of mass, the value Δ_{ME} may be smaller than the actual size of the mesoendodermal layer. Indeed, for cell diameters of the order of the thickness of the mesoendermal layer, the space accessible to the cell center of mass is smaller than the actual layer thickness.

The motion of cells is assumed to alternate between run and tumble phases (see Fig. S4). During the tumble phases the cells perform a non-directed random motion, with the center of mass

$\mathbf{r}(t)$ evolving according to

$$\dot{\mathbf{r}}(t) = \sqrt{2D_t}\boldsymbol{\xi}(t), \tag{9}$$

where D_t is the positional noise during the tumbling phase and $\boldsymbol{\xi}$ is a three dimensional random vector with independent, normally distributed and δ-correlated components $\langle \xi_i(t)\xi_j(t')\rangle = \delta_{ij}\delta(t-t')$. Such a motion is analogous to overdamped Brownian motion [41,42].

In the run phases, cells perform a noisy directed migration. The corresponding Langevin equation of motion for the position $\mathbf{r}(t)$ reads:

$$\dot{\mathbf{r}}(t) = s(t)\mathbf{p}(t) + \sqrt{2D_r}\boldsymbol{\xi}(t), \tag{10}$$

where the first term correspond to directed motion of the cell with speed $s(t)$, along a time-dependent polarization vector $\mathbf{p}(t)$ ($|\mathbf{p}| = 1$). The second term corresponds to a positional noise with strength D_r. In both the run and tumble phases, the positional noise accounts for both random displacements stemming from the activity of the cell and possible measurement errors in the detection of the center of mass during tracking.

Both the speed $s(t)$ and polarization direction $\mathbf{p}(t)$ in the run phase are stochastic variables. To represent the stochastic motion, we choose to represent the cell by an active Brownian particle [42,43]. The speed then follows the stochastic equation:

$$\dot{s}(t) = -\gamma s(t) + f_s(t). \tag{11}$$

The first term represents a friction-like term with a constant speed relaxation rate γ, and the second term $f_s(t)$ is a stochastic driving force, which models the stochastic force generation along the polarization direction due to the formation of protrusions. We assume $f_s(t)$ to be a shot-noise process [43,44], consisting of temporal δ-peaks arriving at times t_i with exponentially distributed amplitudes a_i: $f_s(t) = a_i\delta(t - t_i)$. The amplitudes a_i have mean a_f. In this formulation, the shot-noise process f_s represents the random formation of protrusions, while $1/\gamma$ corresponds to the characteristic life time of protrusions. The rate of the shot noise process r_f determines the average time between two random kicks $\langle \Delta t_{ji}\rangle = r_f^{-1}$.

For simplicity we assume that the polarization vector is parallel to the 2d-layer, $\mathbf{p} = (p_x, p_y, 0)^T$. Therefore, $\mathbf{p}$ is fully specified by a polar angle φ, according to $p_x = \cos\varphi$, and $p_y = \sin\varphi$. The polarization angle $\varphi(t)$ fluctuates around a desired direction φ_D:

$$\dot{\varphi}(t) = -\kappa(\varphi - \varphi_D) + \sqrt{2D_\varphi}\xi_\varphi \tag{12}$$

where external and internal sources of fluctuations leading to deviations from the desired direction are represented by the noise term with intensity D_φ. ξ_φ is an uncorrelated white noise, $\langle \xi_\varphi(t)\xi_\varphi(t')\rangle = \delta(t-t')$; in other words, the value of the noise at at time t does not depend on its value at previous times. In equation 12, the desired angle φ_D is constant during each run, and its value is picked at the beginning of a new run at t^*, according to:

$$\varphi_D = \arg(\mathbf{r}_{\text{target}}(t^*) - \mathbf{r}(t^*)) + \eta \tag{13}$$

with $\mathbf{r}_{\text{target}}$ being the center of mass of the target and η being a detection error drawn from a normal distribution with standard deviation ϵ.

Finally, the run and tumble durations are distributed exponentially with average run and tumble times: τ_r,τ_t. Note that τ_r and τ_t are in general different from the experimentally measured run and tumble times, as explained below (section II B).

Numerical integration of the stochastic equations of motion was performed using the simple Euler forward algorithm. The corresponding discretized equations of motion in the run phase read:

$$\mathbf{x}(t+dt) = \mathbf{x}(t) + s(t)\mathbf{p}(t)dt + \sqrt{2D_r dt}\,\boldsymbol{\xi} \tag{14}$$

$$s(t+dt) = s(t) - \gamma s(t)dt + \tilde{f}_s(t) \tag{15}$$

$$\varphi(t+dt) = \varphi(t) - \kappa(\varphi(t) - \varphi_D)dt + \sqrt{2D_\varphi dt}\,\xi_\varphi, \tag{16}$$

where the components of the vector $\boldsymbol{\xi}$ as well as ξ_φ are normally distributed Gaussian random variables. The variable $\tilde{f}_s$ is the time discretized version of the shot-noise process, it takes the value a_f with a probability $r_f dt$ and is zero otherwise. Note, that according to this definition the units of the shot noise increments have the unit of speed and the energy input due to the stochastic driving depends implicitly on the time step dt, which is fixed in all simulations to $dt = 0.1$. Furthermore, in order to avoid constant driving $r_f dt < 1.0$ should hold.

B. Parametrization of the computational model using experimental measurements

We discuss in this section the identification of model parameters such that model trajectories are statistically similar to recorded experimental trajectories.

The position of the target region is not experimentally known, but it is commonly assumed it moves with the enveloping layer (EVL), which spreads over the embryo over the course of epiboly. We therefore take the target velocity to be equal to the average speed of epiboly, $v_{ep} = 1.5\mu m/min$. The directional error ϵ of the run directions is taken to be of the order of the spread of the

protrusions in the high magnification experiments, $\epsilon = 0.2$. We set the vertical confinement for the center of mass of the cell to $\Delta_{ME} = 1\mu m$. The initial distance of a cell to the target in the xy-plane is assumed to be of the order of the embryo radius, $d_0 = 300\mu m$. In addition to initial distance, the relative position of the cells with respect to the target is defined by an initial polar angle θ_0, which is set to $\theta_0 = 0.5\pi$. Thus the vector connecting the initial position of the cell and the initial target position is given by $x_0 = d_0 \cos(\theta_0)$, $y_0 = d_0 \sin(\theta_0)$, $z_0 = 0$. We have verified that our qualitative results and the corresponding conclusions do not depend on the particular choice of these parameters, as long as they are within experimentally realistic values.

The 9 remaining model parameters are listed in Table S1. To identify values consistent with experimental observations, we compare the 8 observables introduced in Section I C obtained from simulated and experimental cell trajectories. We first identified promising parameter regions with reasonable parameter values by a rough manual scan of the parameter space, rejecting all parameter sets where the relative errors for each observable were above 0.25 (see below and Table S2 for a definition of the relative errors). In a second step, we performed a systematic, automated fitting procedure to find the local optimum in the 9-dimensional parameter space. During the optimization procedure, for a given parameter set, we performed 100 independent runs of the model with the same number of cells ($N = 23$) and the same duration ($t = 90$min) as in the experiments. For each run, observables were calculated in the same way as for experimental data, by simulating positional tracking with the same temporal resolution $\Delta t = 1.5$mins and analyzing the resulting trajectories following the same procedure as for experimental trajectories. We used the results of the independent simulations to estimate the mean values and standard deviations of the observables. Assuming normal distribution and independence of the observables, we then calculated the likelihood of the experimental observable vector being drawn from the observable probability distribution estimated from simulations. Finally, this likelihood was maximized using an evolutionary algorithm optimization, the Covariance Matrix Adaptation Evolution Strategy (CMA-ES), which is well suited for noisy optimization problems [45]. The parameters obtained from fitting the model to the wild-type cell data (MZ*oep* host) are shown in Table S1.

Table S2 shows a comparison of the observables calculated from experimental data to the ones obtained from a sample simulation runs with the fitted parameter values. Using the same analysis for trajectories generated by simulations and experimental trajectories, we ensure that T_r and T_t match between experiments and simulations (T_r and T_t, the observed run and tumble times, are defined in section I C). Note that the run and tumble times τ_r and τ_t (the microscopic model parameters) are in general different from T_r and T_t (the measured run and tumble times). Indeed,

parameter	est. value	std. deviation	unit	description
$\tau_{t,exp}$	1.37	0.008	min	average tumble time
$\tau_{r,exp}$	8.00	0.009	min	average run time
D_t	3.83	0.009	$\mu m^2 \text{min}^{-1}$	positional noise in the tumble phase
D_r	0.29	0.005	$\mu m^2 \text{min}^{-1}$	positional noise in the run phase
D_φ	0.16	0.009	$\text{rad}^2 \text{min}^{-1}$	angular noise of the polarization direction
a_f	0.94	0.006	$\mu m\, \text{min}^{-1}$	amplitude of stochastic driving force
r_f	1.97	0.008	min^{-1}	rate of stochastic driving force during runs
γ	1.41	0.009	min^{-1}	speed relaxation rate during runs
κ	1.17	0.010	min^{-1}	relaxation rate of polarization direction

TABLE S1: Estimated parameters and their standard deviations obtained using the CMA-ES algorithm maximizing the likelihood of a match between experimental and simulation data. The optimization was terminated once the estimated standard deviation for all parameters was $\leqslant 0.01$.

the latter are calculated from measured trajectories and depend on details of the trajectories measurement, such as the temporal tracking resolution. τ_r and τ_t on the other hand represent the duration of the "internal" states of a cell in our model, which are not directly accessible through tracking. Due to false positive tumble detection along the noisy cell trajectory, it is expected that the direct estimate of the run time from tracking data systematically underestimates the run duration $T_r \lesssim \tau_r$, consistent with results of the simulations. We also observe an overestimation of the measured tumble durations $T_t \gtrsim \tau_t$, which is likely to originate from the coarse temporal resolution.

To further test our fitting procedure, we compared the speed distributions conditioned on the state (run/tumble) and the two dimensional distribution $P(A, S)$ obtained from experiments with those obtained from simulations using the fitted parameter sets. The run/tumble speed distributions show a very good qualitative and quantitative agreement between theory and experiment (Fig. 5E). The probability distribution $P(A, S)$ obtained from simulations also has the same shape as the probability distribution obtained from experimental data (Figs. 5C,D). The shape of the distribution $P(A, S)$ at low alignment values may vary significantly between different simulation runs due to the small number of observations from 23 tracks; however the general pattern recapitulates well the experimental observations.

Overall, the migration model reproduces faithfully the statistical properties of experimental trajectories. An comparison between experimental and simulated trajectories is shown in Supplementary Figure S5.

observable	experimental value	avg. simulation value	avg. rel. error
$\langle\lvert\bar{\mathbf{v}}\rvert\rangle_{\mathrm{t}}$	0.95	0.90	0.05
$\langle\lvert\bar{\mathbf{v}}\rvert\rangle_{\mathrm{r}}$	1.73	1.70	0.02
$\langle\lvert\Delta\mathbf{v}\rvert\rangle_{\mathrm{t}}$	1.30	1.30	< 0.01
$\langle\lvert\Delta\mathbf{v}\rvert\rangle_{\mathrm{r}}$	0.83	0.80	0.03
$\langle\overline{\lvert\mathbf{v}\rvert}\rangle_{\mathrm{t}}$	1.56	1.51	0.03
$\langle\overline{\lvert\mathbf{v}\rvert}\rangle_{\mathrm{r}}$	1.87	1.81	0.03
T_t	3.11	3.09	< 0.01
T_r	5.01	4.98	< 0.01

TABLE S2: Comparison of the observables extracted from experimental and simulated trajectories for the fitted parameters given above. The average simulation values and average relative error between a single simulation run and the experimental result were obtained by averaging over 100 independent simulation runs. Each run was performed with same number of trajectories as in the experiment (N=23). All velocities are in units μm/min; all times in min.

C. Model Predictions - Distance to Target and Positional Variance

We then used our computational model with the parameters estimated from fitting the simulation results to experimental data (wt mesendodermal cells, MZ*oep* host), to systematically explore the effect of changing the average run time on directed cell migration. Numerical simulations were performed on a time $t_e = 90$min, taken equal to the time of experimental observations of cell migration. Note that with the choice of parameters we explored, this time is not long enough for the cells to reach or overshoot the target position. We quantify the migration behavior of individual cells using:

1. the average distance to the target region at the end of the simulation:

$$d(t_e) = \langle \mathbf{r}_i(t_e) - \mathbf{r}_{target}(t_e) \rangle$$

2. the positional variance

$$\sigma^2(t_e) = \left\langle (\mathbf{r}_i(t_e) - \langle \mathbf{r}_i(t_e) \rangle)^2 \right\rangle$$

Here $\langle \cdot \rangle$ indicates the ensemble average over all simulated cells i ($i = 1, \ldots, N$).

We then calculated the average values of $d(t_e)$ and $\sigma^2(t_e)$ as a function of τ_r, keeping all other parameters as in Table S1, for N=100 simulations (Fig. 5B). We find that the average distance to target $d(t_e)$ is decreasing with increasing average run duration τ_r. Indeed, faster cell migration is

achieved for higher run times, as the time spent in the directed run phase increases in comparison to the time spent in the undirected tumbling phase. However with increasing τ_r, the value of $d(t_e)$ appears to stall (Fig. 5B): too large run times indeed lead to a loss of precision in migration, as the cell does not stop frequently enough to accurately track the target.

The variance of the cell positions $\sigma^2(t_e)$ shows a non-monotonic behavior: initially, it decreases with increasing τ_r, but reaches a minimum at finite ratios of run to tumble times. For parameter values explored in the simulations, the minimum is located in the vicinity of the run time obtained from fitting the model to experimental data $\tau_r/\tau_{r,exp} \approx 0.5 - 2$ (Fig. 5B and Fig. S4).

There is therefore a trade-off between speed (minimizing d) and accuracy (minimizing σ^2) for cells migrating using run and tumbles towards a moving target. For large τ_r/τ_t, faster cell migration comes at the expense of a larger spread of the cell population. This result is independent on the model details as well as detailed choice of parameters, and can be observed also in simpler idealized models of run and tumble migration [31].

In Fig. 5B, the shaded region indicates the region where τ_r is consistent with experimental observations. It is the range of average run times, where the mean difference of observables between experiment and model is below 0.1 (Fig. S4). Thus our results suggest that the run and tumble ratio observed in mesoendodermal progenitor cells is close to the optimal one, with respect to the speed versus accuracy trade-off.

Finally we matched the measured T_r/T_t ratio for CA*Ezrin* and *ezrin*-MO to simulated data, assuming that all other parameters remained unchanged and thus estimated the values of the microscopic model parameter τ_r for these two conditions. Examples of the resulting trajectories are shown in Figure S5 and exhibit larger cell dispersion as shown in Fig. 5B.
