# Supplementary figures and images for: Steering cell migration by alternating blebs and actin-rich protrusions
Source: BMC Biol. 2016 Sep 2;14(1):74. doi: 10.1186/s12915-016-0294-x (PMC5010735; doi:10.1186/s12915-016-0294-x)

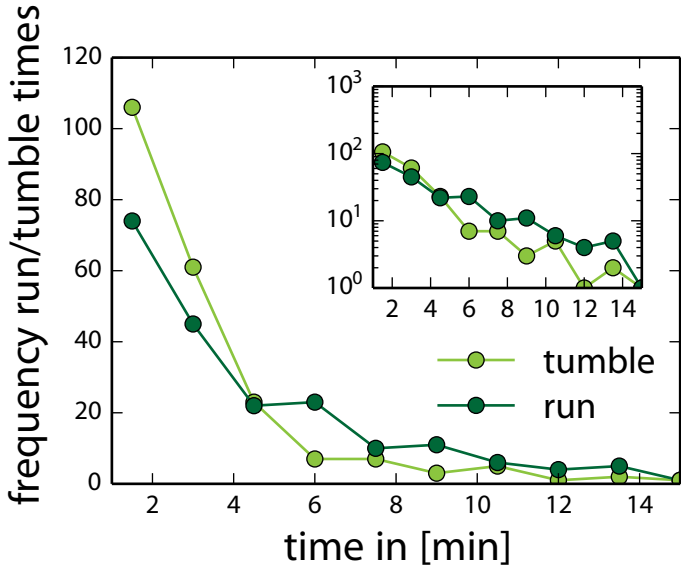

Supplement: Additional file 2: Figure S1. — Durations of run and tumble phases. Distribution of run and tumble durations as detected by our algorithm from low-magnification movies of wt cells transplanted in a MZoep host. Frames were captured at 90 s intervals for 3 h (~5.5–8.5 hpf). The inset shows the same data in a semi-logarithmic plot, where a line indicates an exponential relationship. Exponentially distributed times are expected in a run and tumble trajectory if we assume a simple two-state Markov processes, namely a process that undergoes transitions from one state to another where the probability distribution of the next state depends only on the current state and not on the sequence of events that preceded it [41]. (PDF 103 kb) [file 12915_2016_294_MOESM2_ESM.pdf]

A

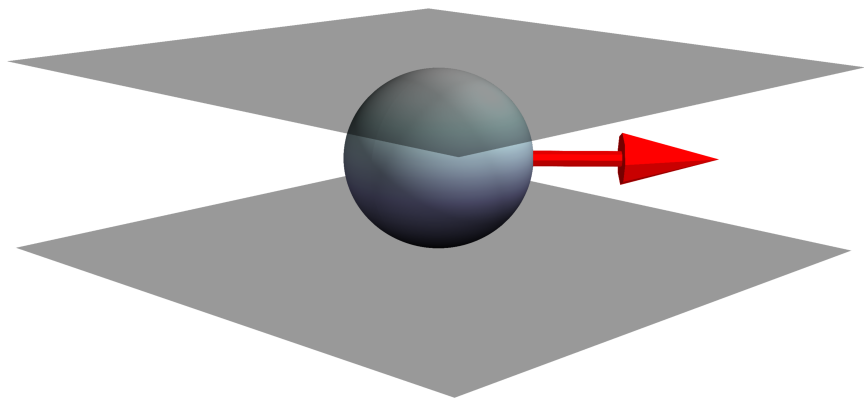

B

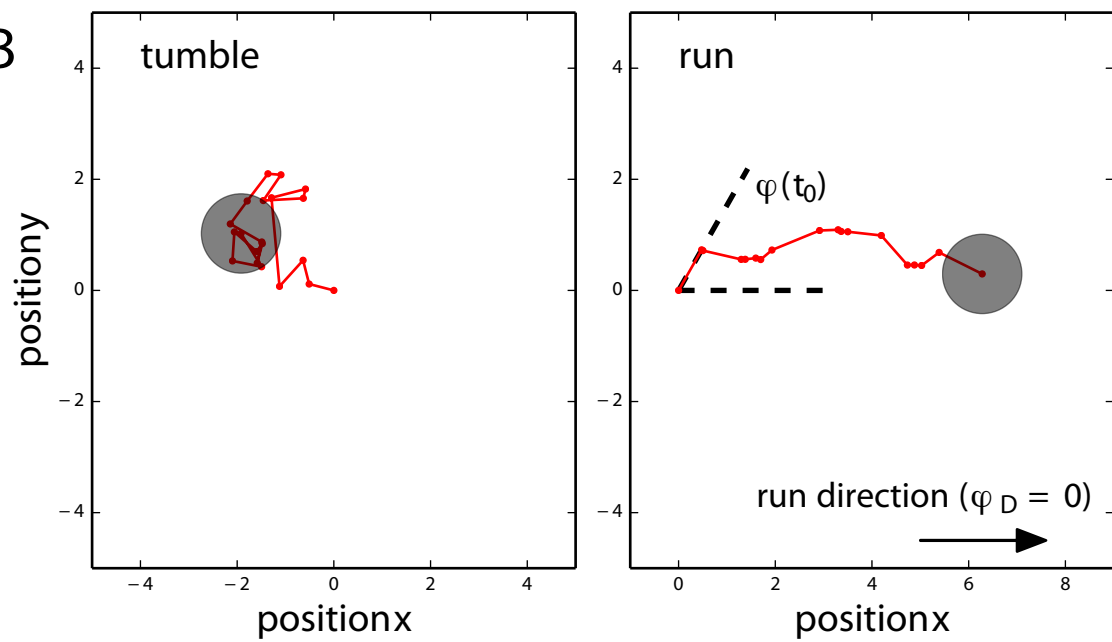

C

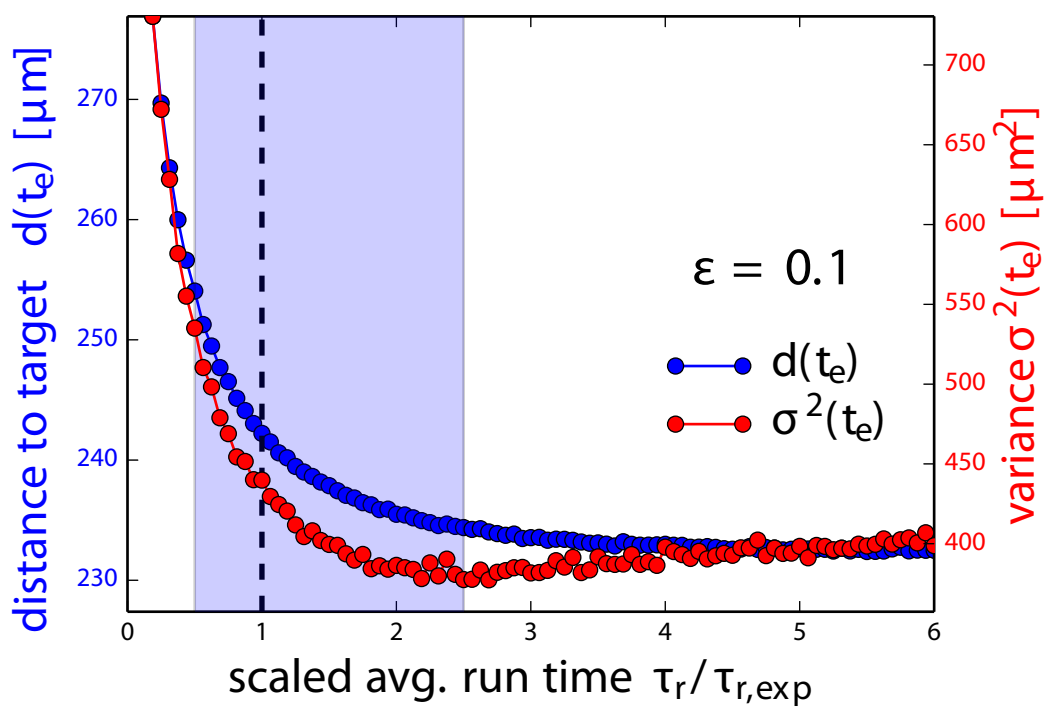

D

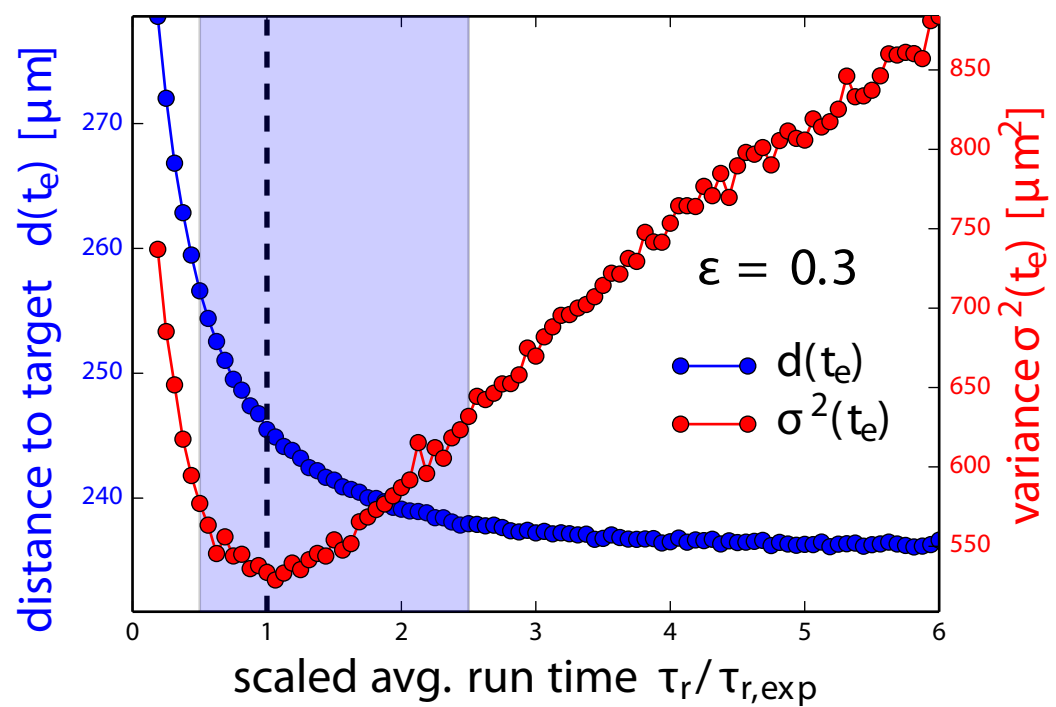

E

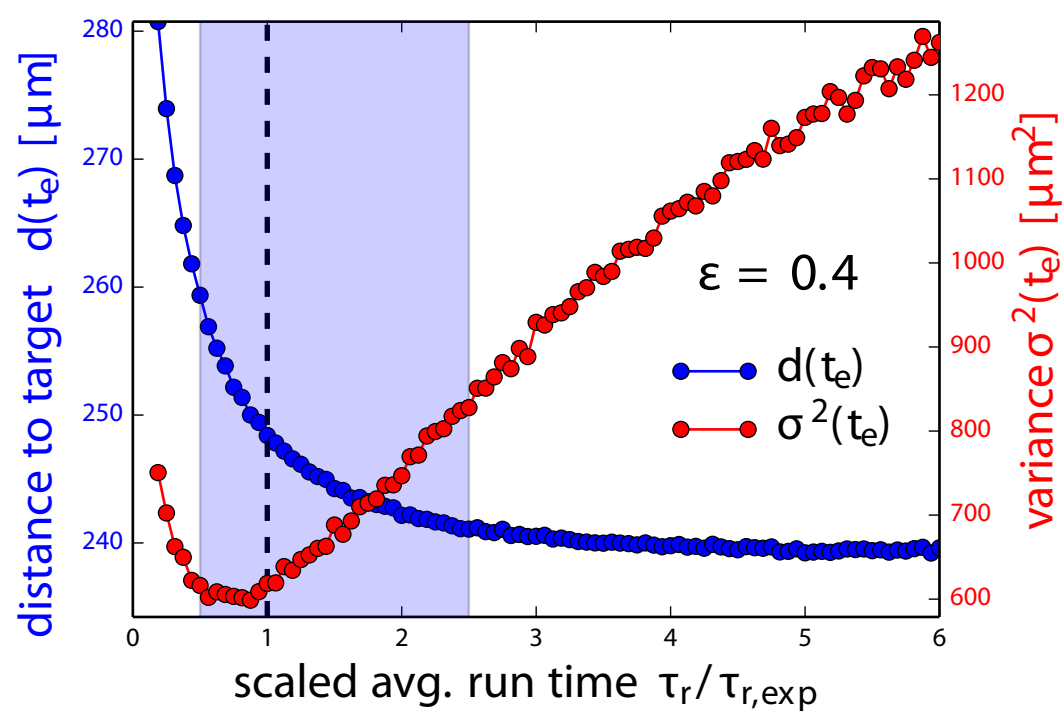

F

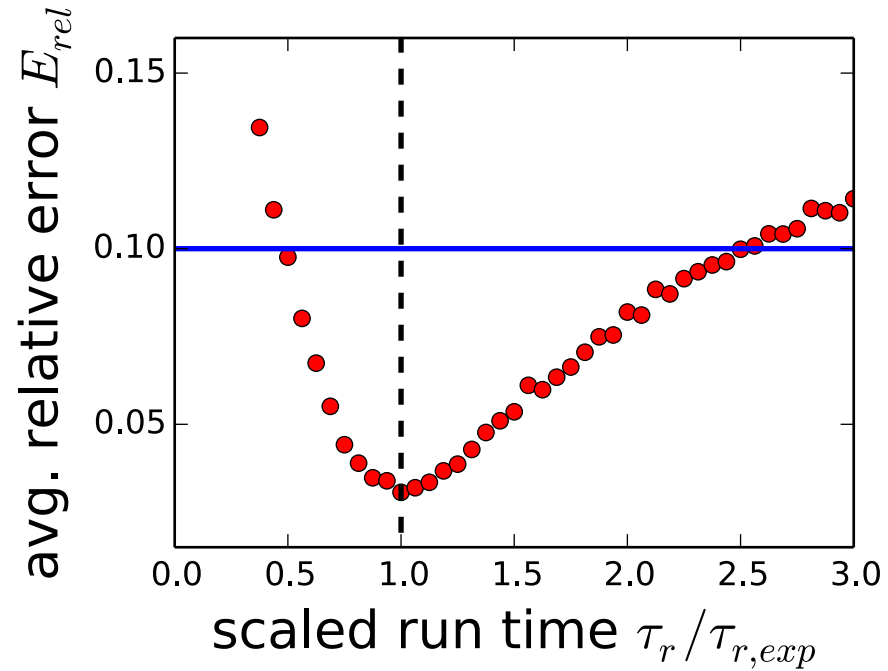

Supplement: Additional file 10: Figure S4. — Computational model description and supplementary results. (A) Schematic visualization of the simulation set-up: three-dimensional model where the cell is confined in the z-direction to the mesoendodermal layer of height ΔME. The red arrow indicates the direction of polarization within the layer. (B) Schematic visualization of example model trajectories in the mesoendodermal plane (xy) in the tumble and run phases. The arrow in the run panel indicates the direction of the run determined by the polar angle 'φD = 0, which sets the average direction of migration during the run. The dashed lines indicate the instantaneous movement angle 'φ(t0) at time t0. Impact of orientation error ε on the model results (C–E). Distance to target and spatial variance of the cells at t = 90 min as a function of the scaled avg. run time τr for different values of the reorientation error towards the target ε (value estimated for wt cells and used in the main text ε = 0:2, Fig. 5); (C) ε = 0:1, (D) ε = 0:3, and (E) ε = 0:4. Average relative error versus run time (F). Average relative error E rel defined as the mean observable deviation (Additional file 1: Table S2) between model simulations and experimental results (wt cells in MZoep host). All model parameters as fitted to experimental data with the average run time being varied around the fitted value as in Fig. 5b. The vertical line represents the threshold value 0.1, which defines the shaded ‘consistency region’ in Fig. 5b. (PDF 1475 kb) [file 12915_2016_294_MOESM10_ESM.pdf]

A

experiments

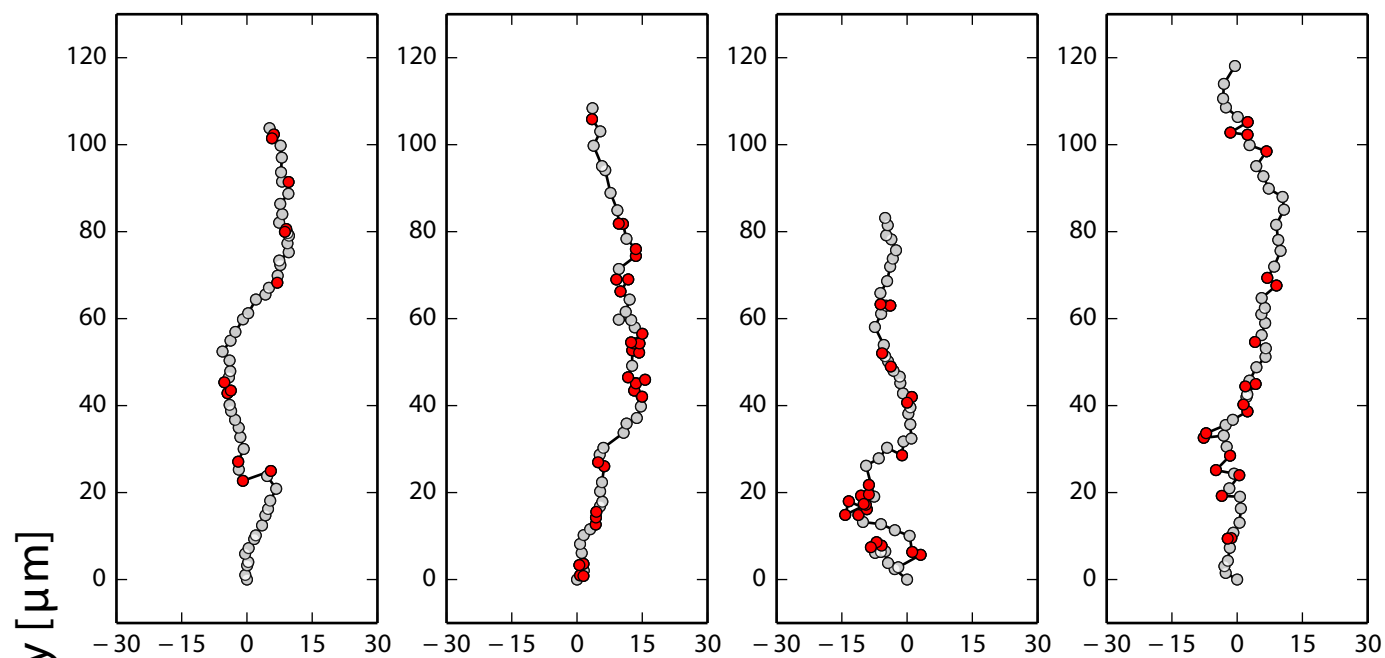

simulations

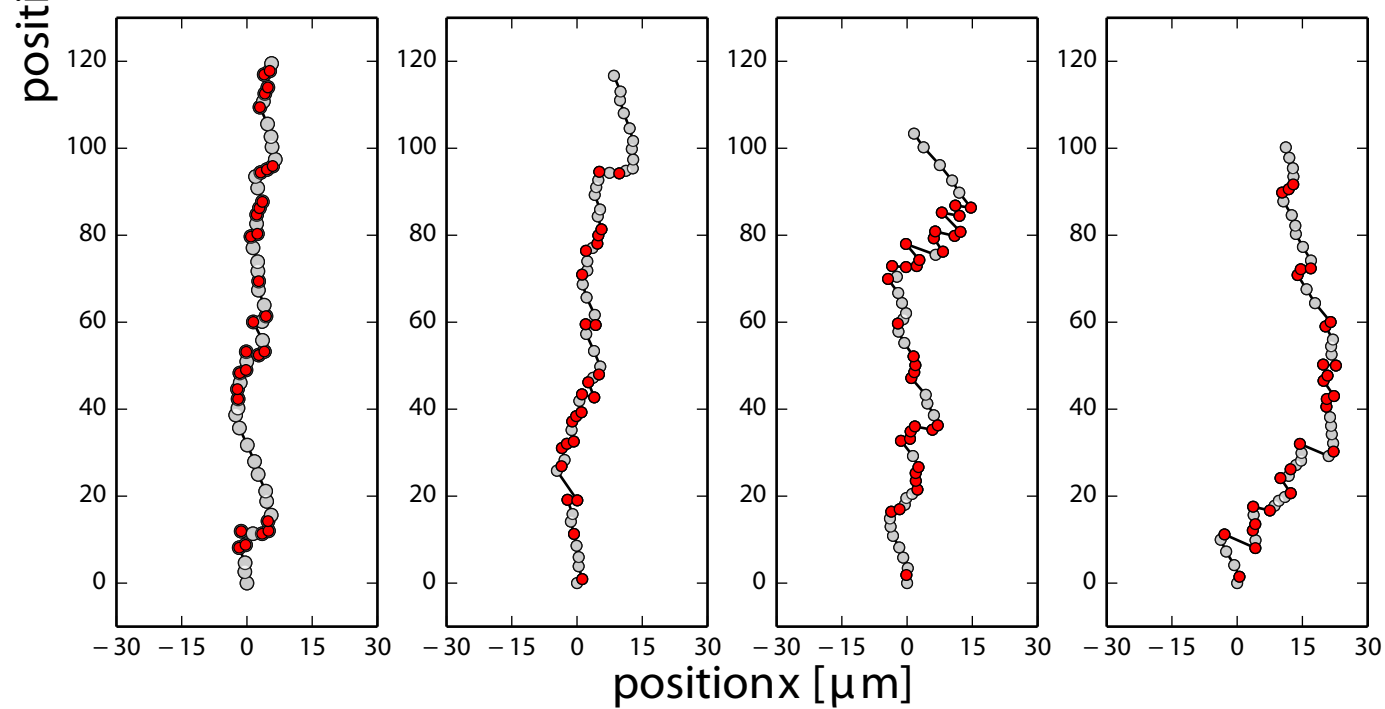

B

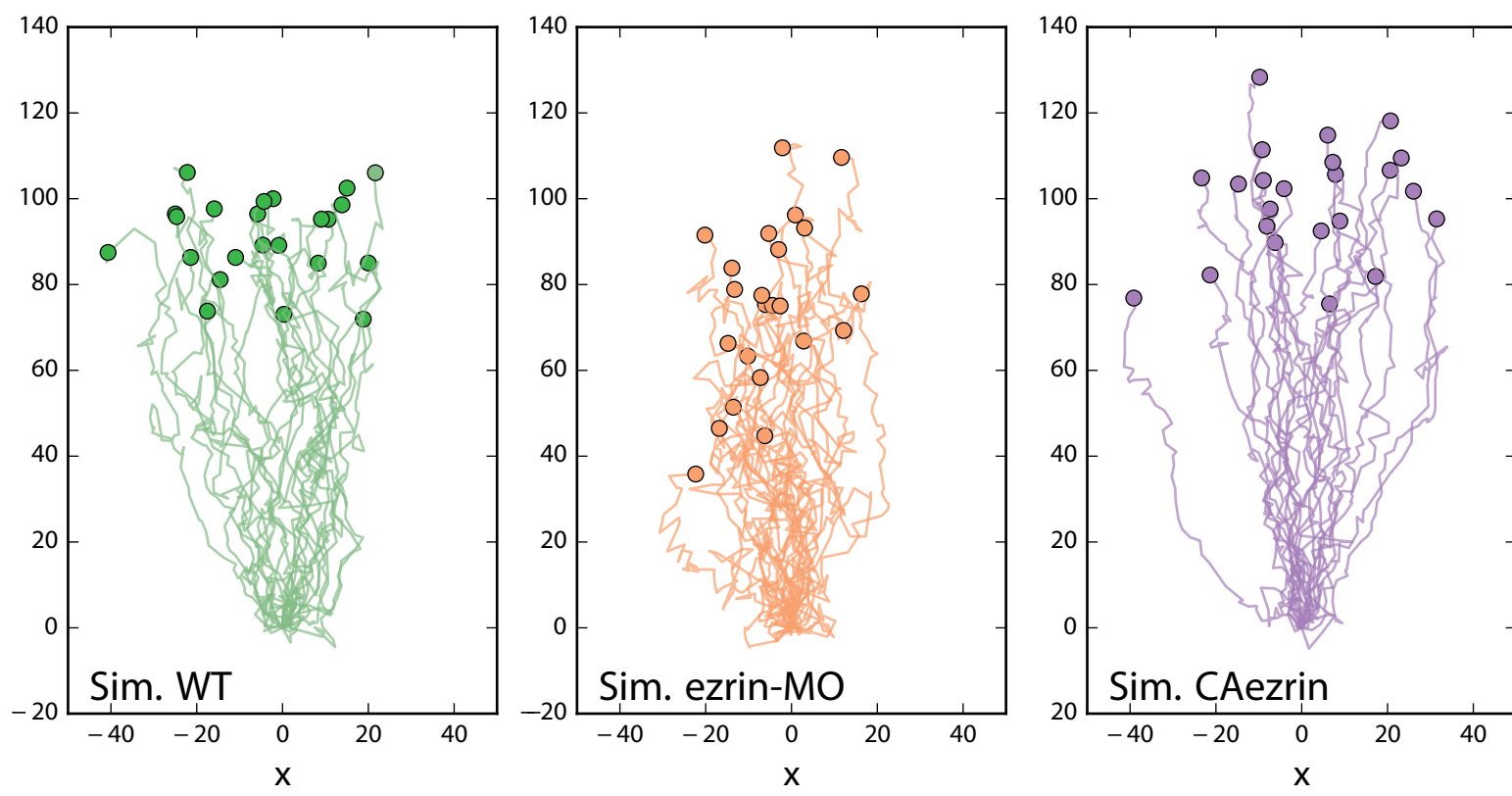

Supplement: Additional file 11: Figure S5. — Comparison of experimental and simulated cell trajectories (A). Examples of cell trajectories from wild-type cells in an MZoep host from low-magnification experiments (top) and from model simulations with the fitted parameters (bottom). All experimental (three-dimensional) trajectories were projected to the primary two-dimensional plane of their motion and rotated so that their average direction of migration aligns with the y-axis as in the simulations. Frames were captured at 90 s intervals for 3 h (~5.5–8.5 hpf). The red symbols indicate the points along the trajectory identified as tumbling events using the algorithm introduced in Sec. I C. Simulated cell trajectories corresponding to different experimental conditions (B). Examples of simulated cell trajectories for different values of model parameter τr with other parameters as fitted for wt cells in an MZoep embryo. (left) τr as fitted to the wt cells (τr = 8 min). (center) For long runs (τr = 25 min), which yield T r = T t - values consistent with results obtained from CAEzrin cells, and (right) short runs (τr = 4 min), which yield T r = T t, values consistent with the ones measured for ezrin-MO cells. (PDF 1651 kb) [file 12915_2016_294_MOESM11_ESM.pdf]
