# Supplementary material for: Steering cell migration by alternating blebs and actin-rich protrusions
Source: BMC Biol. 2016 Sep 2;14(1):74. doi: 10.1186/s12915-016-0294-x (PMC5010735; doi:10.1186/s12915-016-0294-x)
Supplement: Additional file 4: Figure S2. — Accuracy of protrusion detection using the Automatic Protrusion Analyzer (APA). APA accurately detects 83 % of the blebs formed by the cells as validated by two independent experimentalists. The majority of false positive detected blebs consisted of the same bleb being detected at more than one time point. Number of analyzed cells = 28. Manual control was also performed for actin-rich protrusions and detection was found to be accurate for more than 99 % of the protrusions and for this reason we do not provide the results of the manual segmentation here. (PDF 148 kb) [file 12915_2016_294_MOESM4_ESM.pdf]

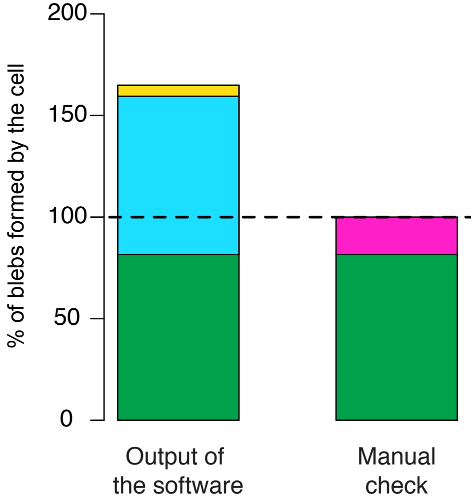

- False negatives: Blebs detected by eye but not by the software
- Detected more than once: APA detected the same bleb in more than one timepoint or twice in the same timepoint
- False positives: APA detected a cell deformation that was not a bleb.
- Blebs detected both manually and by APA
