# Supplementary material for: Steering cell migration by alternating blebs and actin-rich protrusions
Source: BMC Biol. 2016 Sep 2;14(1):74. doi: 10.1186/s12915-016-0294-x (PMC5010735; doi:10.1186/s12915-016-0294-x)
Supplement: Additional file 7: Figure S3. — Frequency of actin-rich protrusions, orientation of cell protrusions, and summary of POP values. (A) Frequency of actin-rich protrusions in control and ezrin-MO-injected mesendoderm cells. Arbitrary units (AU) are used as actin-rich protrusions are weighted with the intensity of the Lifeact signal in the protrusion. (B) Orientation of blebs with respect to the local migration axis. POP: Mean ± SEM of the magnitude of the polar order parameter. Number of analyzed cells = 17 for wt, 6 for ezrin-MO, and 6 for CAEzrin. Number of blebs n = 349 for wt, 163 for ezrin-MO, and 6 for CAEzrin. Statistical significance was determined comparing the mean ± SEM of the magnitude of the POP of the angular distributions. (C) Orientation of bleb and actin-rich protrusion formation with respect to the Yolk-ectoderm axis. For all experimental conditions, protrusions are almost exclusively oriented perpendicular to the Yolk-ectoderm axis, indicating that protrusions are formed into the extracellular space between the Yolk cell and the overlaying ectoderm layer. (D) Mean values ± SEM of the magnitude of the POP of the angular distributions of the analyzed protrusions. Green shaded area covers the wt mean ± SEM. Number of analyzed cells = 17 for wt, 6 for ezrin-MO, and 6 for CAEzrin. Number of actin-rich protrusions = 10853 for wt, 1501 for ezrin-MO, 1160 and 2549 for CAEzrin. Number of blebs n = 349 for wt and 163 for ezrin-MO. For CAEzrin only 6 blebs were observed so the POP was not calculated. (PDF 600 kb) [file 12915_2016_294_MOESM7_ESM.pdf]

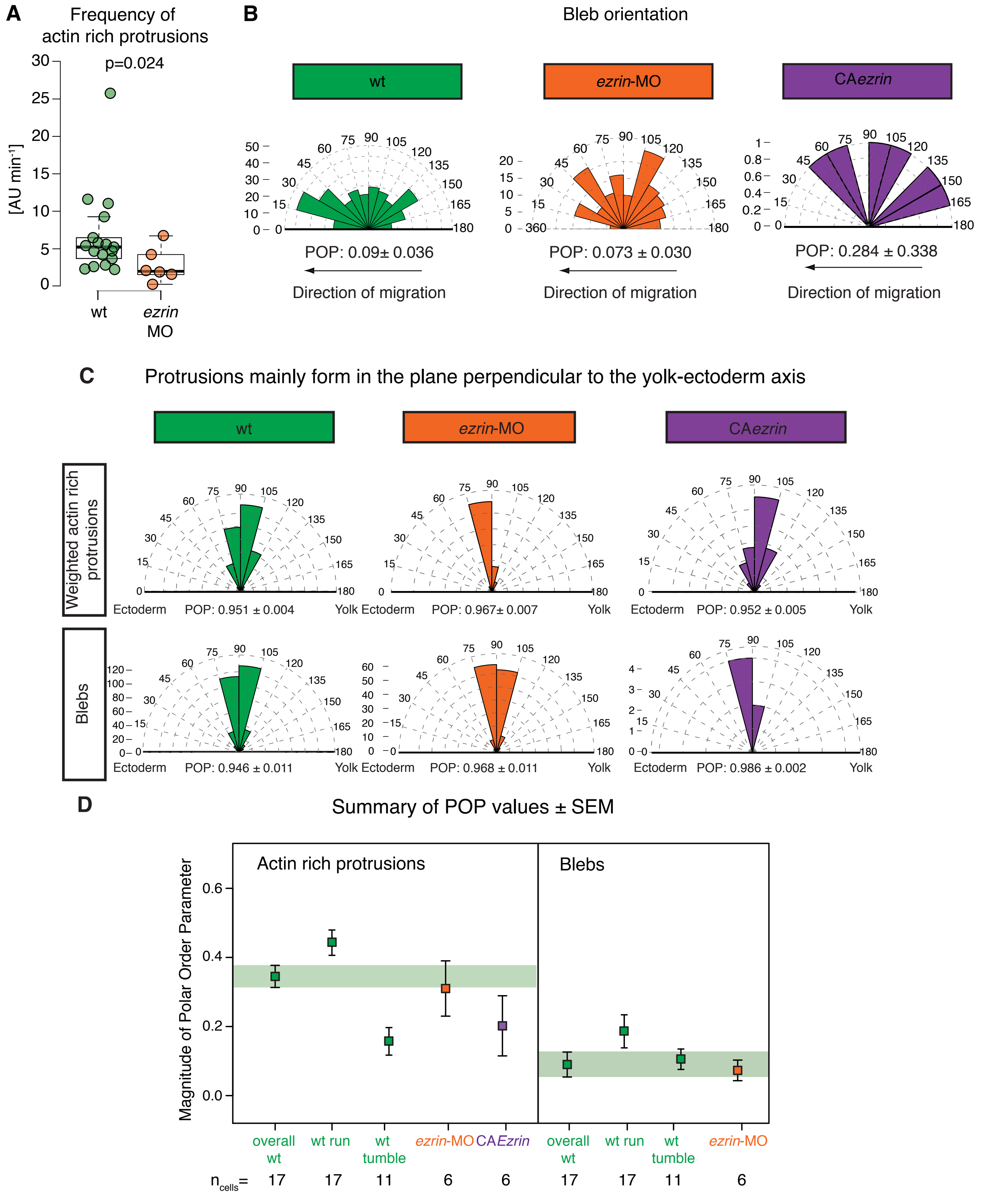

A
Frequency of actin rich protrusions
p=0.024
[AU min-1]
wt
ezrin MO
B
Bleb orientation
wt
ezrin-MO
CAezrin
POP: 0.09± 0.036
POP: 0.073 ± 0.030
POP: 0.284 ± 0.338
Direction of migration
C
Protrusions mainly form in the plane perpendicular to the yolk-ectoderm axis
Weighted actin rich protrusions
Blebs
Ectoderm
Yolk
POP: 0.951 ± 0.004
POP: 0.967± 0.007
POP: 0.952 ± 0.005
POP: 0.946 ± 0.011
POP: 0.968 ± 0.011
POP: 0.986 ± 0.002
D
Summary of POP values ± SEM
Actin rich protrusions
Blebs
Magnitude of Polar Order Parameter
overall wt
wt run
wt tumble
ezrin-MO
CAEzrin
n cells= 17 17 11 6 6 17 17 11 6
